# Distractor avoidance is achieved by active control rather than passive exclusion

**DOI:** 10.3758/s13414-026-03317-5

**Published:** 2026-08-03

**Authors:** Xiaojin Ma, Richard A. Abrams

**Affiliations:** 1 Department of Psychological & Brain Sciences, Washington University in St. Louis, St. Louis, MO USA; 2 Department of Psychological Sciences, University of Missouri, Columbia, MO USA

**Keywords:** Visual attention, Visual search, Attentional capture, Attentional suppression, Inhibitory control

## Abstract

**Abstract:**

When navigating the visual world, we have considerable control over what stimuli to select for deeper processing, and we can often resist capture by physically salient but task-irrelevant distractors. A debate has emerged regarding whether distractor avoidance is accomplished by active goal-driven attentional control, or by passive exclusion of distractors from the narrow attentional focus of a highly serial search, as the *attentional window account* claims. In the present study, in order to explore the issue, we measured the extent to which attentional avoidance of salient distractors is affected by the seriality of the search. In three experiments, we invoked different levels of seriality by separately manipulating the similarity among search elements, the stimulus pattern complexity, and the conjunctive features that defined the target. To assess attentional allocation, probe trials were interleaved with the search trials. While the results showed significant avoidance of color singleton distractors in all experiments, the strength of the avoidance effect, as measured by both search latency and probe report, did not differ as a function of the seriality of the search. An additional control experiment verified the item-by-item serial search processes invoked by most of our methods. The results are inconsistent with the passive filtering proposed by the attentional window account and instead imply that active top-down control is a more likely explanation for distractor avoidance.

**Open practices statement:**

Trial-level data and analysis code for all experiments have been made publicly available online (https://osf.io/njvu2), and none of the experiments was preregistered.

Salient items in a visual scene can capture our attention. However, when such items are not relevant to our current goals, we are able to ignore them. This is exemplified by our ability to keep our eyes (and attention) on the road when driving at night despite the potentially distracting lights from the dashboard. Because the ability to avoid distraction is a fundamental part of efficient visual processing, researchers have devoted considerable effort to learning more about details of the underlying mechanisms. We continue that endeavor here by explicitly testing two broadly different types of explanations of our ability to ignore salient distractors.

According to one explanation for distractor ignoring, attention can be guided in a top-down manner to favor task-relevant items and avoid irrelevant ones. A large body of research has demonstrated that goal-matching features receive prioritized processing over distractors that lack relevant features (Becker et al., 2010; Folk et al., 1992, 2002; Lien et al., 2008). Moreover, a recent theory proposed that our ability to prevent attentional capture by salient-but-irrelevant distractors is also due to goal-driven control, in which the feature that signals salience is perceptually downweighted (the *signal suppression hypothesis*; Gaspelin & Luck, 2018c, 2019; Luck et al., 2020; Sawaki & Luck, 2010). The signal suppression hypothesis has received support from multiple sources. In behavioral studies, using a probe task that measures the allocation of attention during the initial exposure to a visual display, it was found that probes on salient task-irrelevant color singleton distractors had lower report rates than probes on nonsalient nontarget items (Chang & Egeth, 2021; Gaspelin et al., 2015; Ma & Abrams, 2023b, c; Stilwell & Gaspelin, 2021). Similarly, initial saccades following stimulus onset were found to be less frequently directed to a salient compared with a nonsalient distractor (Drennan & Gaspelin, 2026; Gaspelin et al., 2017, 2019; Ipata et al., 2006; H. Zhang et al., 2024). Measurements of event-related potentials show that such behavioral avoidance of salient distractors is often preceded by a positive waveform with a distribution contralateral to the distractor location—a distractor positivity (P_D_) component, thought to index inhibitory control (Gaspelin, Lamy, et al., 2023; Gaspelin & Luck, 2018a; Gaspar & McDonald, 2014; Hickey et al., 2009; Jannati et al., 2013; Sawaki & Luck, 2010). In those studies, the observed effects of distractor avoidance may reflect a combination of distractor inhibition and other forms of attentional guidance (e.g., target feature enhancement, biased competition; Chang & Egeth, 2019; Desimone & Duncan, 1995; Gaspelin et al., 2025; Khodayari et al., 2026; Wolfe, 2021). Regardless of the specific mechanism, these effects all rely on top-down knowledge about the task goals, and thus broadly support the existence of goal-driven control mechanisms that actively prioritize goal-relevant features and meanwhile inhibit capture by salient, irrelevant features.

In contrast, another viewpoint, the *attentional window account*, rejects a role of active goal-driven control but attributes the absence of capture by salient distractors as a nonvolitional consequence of narrowly focused attention. Specifically, the theory claims that a relatively small area of a scene is selected (i.e., a small “attentional window”) at any one time during a serial search that requires fine discrimination (Theeuwes, 2004, 2023). Salient stimuli outside the attentional window do not capture attention—not because they are actively avoided in a goal-directed manner but instead because their salience signals are passively excluded from spatial selection and thus fail to propagate to later stages of processing. The key distinction between this account and goal-driven theories, therefore, is whether distractor ignoring reflects active control (guiding attention away from irrelevant features) or passive exclusion (shielding unattended items from selection). Evidence supporting the attentional window account showed that distractor ignoring has only been observed during searches for a nonsalient, prespecified target shape among heterogeneous distractors (a *specific-target* task), where attention needs to be relatively narrowly focused (i.e., *feature-search mode*; Chang & Egeth, 2021; Gaspelin et al., 2015, 2017; Stilwell & Gaspelin, 2021), but not in a search for a unique, and thus salient, target shape among homogeneous distractors (an *additional singleton* task), where attention is spread more broadly (i.e., *singleton-detection mode*; Theeuwes, 1992, 1994, 2004).

There is considerable support for the general ideas underlying the attentional window account, which is rooted in the classical works on attentional guidance. The idea that attention can be spread at different breadth under different task requirements is also supported by previous literature (Eriksen & St. James, 1986; LaBerge, 1983; Leonard et al., 2013). For example, LaBerge (1983) had participants either attend to an entire word or the middle letter of a word, while performing a simultaneous probe identification task. Probes that appeared at different locations of the word were identified with equal accuracy in the attend-word condition, but in the attend-letter condition accuracy appeared to show a gradient with a peak at the location of the attended letter. In that study, different breadths of attentional focus were directly invoked by the instructions—in other cases, they may be indirectly invoked by implicit task demands. Neisser (1967) introduced the idea of different modes of attentional control in visual search tasks, which was later characterized as a distinction between serial searches (in which the target item can only be found by inspecting items one at a time) and parallel searches (in which the target item can be found by scanning all items in a scene simultaneously; Duncan & Humphreys, 1989; Egeth et al., 1984; Kaptein et al., 1995; Theeuwes, 2004). Treisman and Gelade (1980) further illustrated that the search mode is contingent on the requirements of the task: An increased need for feature binding in target discrimination would lead to a more serial search process. These classical findings establish the basic premise underlying the attentional window account—that attention can be spread at different breadths, and the requirement of fine discrimination during a search can cause attention to be narrowly focused.

Despite support for the underlying theory, the attentional window account makes specific and untested predictions when being applied to explain distractor ignoring. It predicts that when salient distractors cause no disruption, search must have been highly serial, preventing even initial selection of the salient item. Some studies support it to an extent, with the level of seriality indexed by the slopes of reaction time (RT) as a function of the number of elements in the search array (i.e., set size; Jonides & Yantis, 1988; Pashler, 1987; Theeuwes, 2004). Very shallow slopes are thought to be indicative of parallel searches, whereas steep ones indicate a serial search. For example, Theeuwes (2004) found flat slopes (indicative of parallel processing) in an additional singleton task (in which capture by a singleton distractor was observed), but positive slopes (indicative of serial or partially serial processing) in a specific-target task (in which there was no capture). However, because this comparison involved two distinct task types rather than a direct manipulation of search seriality, it provides only correlational evidence linking distractor ignoring to serial search. Meanwhile, other researchers have argued against using search slopes to index seriality (e.g., Liesefeld & Müller, 2019; Michaelsen et al., 2024; Palmer, 1995; Townsend, 1971). Other aspects of search behavior indicate that searches that permit distractor ignoring are not strictly serial, because the search target is highly likely to be promptly attended as soon as the display is presented: Probe results from several studies showed significantly higher report rates for target probes than nonsingleton distractors (Gaspelin et al., 2015; Ma & Abrams, 2023c; Stilwell et al., 2023; Stilwell & Gaspelin, 2021). Eye-tracking studies also showed that the first saccades landed on the target significantly above chance (Gaspelin et al., 2017, 2019). Such rapid target selection implies substantial parallel processing before the first attentional shift, which is difficult to reconcile with a narrow attentional window.

Thus, existing studies provide mainly correlational evidence linking search seriality to distractor ignoring. To more directly test the attentional window account, the present study systematically varied search seriality within a given search task and examined its effect on the ability to ignore salient distractors. Specifically, we created highly serial search conditions that, according to the theory, should induce a narrow attentional window capable of spatially filtering salient distractors. Thus, the attentional window account would predict stronger filtering of salient distractors in more serial search conditions, while the goal-driven account would predict that distractor ignoring would not vary with search seriality.

The seriality of search is conventionally manipulated by varying the physical similarity between target and distractors in a search array (Gaspelin et al., 2016; Kerzel & Huynh Cong, 2024; Ruthruff et al., 2020). Several studies have manipulated this factor and examined its impact on ignoring a salient color-singleton distractor. Ruthruff et al. (2020) had participants search for a circle target among oval distractors, with target–distractor similarity manipulated by varying the circularity of the ovals. Similarly, Kerzel and Huynh Cong (2024) also manipulated target–distractor similarity by varying geometric attributes of the distractors: A square target was surrounded by circles in the low-similarity condition and by diamonds that were also angular in the high-similarity condition. Both studies found lengthened RT as target–distractor similarity increased, verifying that the search did become more serial. Despite that, when the task required a feature-search mode, both studies showed that there was no effect of increased seriality on distractor ignoring. And when the task required a singleton-detection mode, higher search seriality led to greater capture by a distractor—opposite to the prediction of the attentional window account. These studies provided initial evidence refuting the attentional window account. However, they only show the elimination of performance costs associated with a salient distractor—not below-baseline distractor avoidance. Thus, they do not provide strong support for goal-driven distractor avoidance. In addition, there are a number of other ways to manipulate search seriality, and employing such alternatives would permit greater generalization of the conclusions to different visual contexts and task requirements.

The present study employed a wide variety of methods to vary search seriality and test the attentional window account. Importantly, in contrast to Kerzel and Huynh Cong (2024) and Ruthruff et al. (2020), the present experiments used search conditions that are known to permit active avoidance of salient distractors, in order to compare the strength of the inhibition effect across different levels of search seriality. In separate experiments, we used three different methods to manipulate target–distractor discriminability, and the impact of a salient, task-irrelevant color singleton distractor was examined. In Experiment 1, we manipulated the physical resemblance between search array items (as examined by earlier researchers). In Experiment 2 we altered stimulus discriminability by manipulating the stimulus pattern complexity (as in Sun & Firestone, 2021). And in Experiment 3, we manipulated search seriality not by altering bottom-up factors but instead by varying the top-down demands of the task in a conjunction search (as in Egeth et al., 1984; Treisman & Gelade, 1980; Zohary & Hochstein, 1989). An additional control experiment showed evidence that the search was indeed more serial under most of our manipulations, but the extent to which a salient distractor was inhibited did not differ. This violates the prediction of the attentional window account, which claims that distractor avoidance should be enhanced when the search becomes more serial. Instead, the results are consistent with goal-driven mechanisms that guide attention to favor relevant items and avoid irrelevant items, and can do so regardless of the seriality of the search.

## Experiment 1

**Fig. 1:**
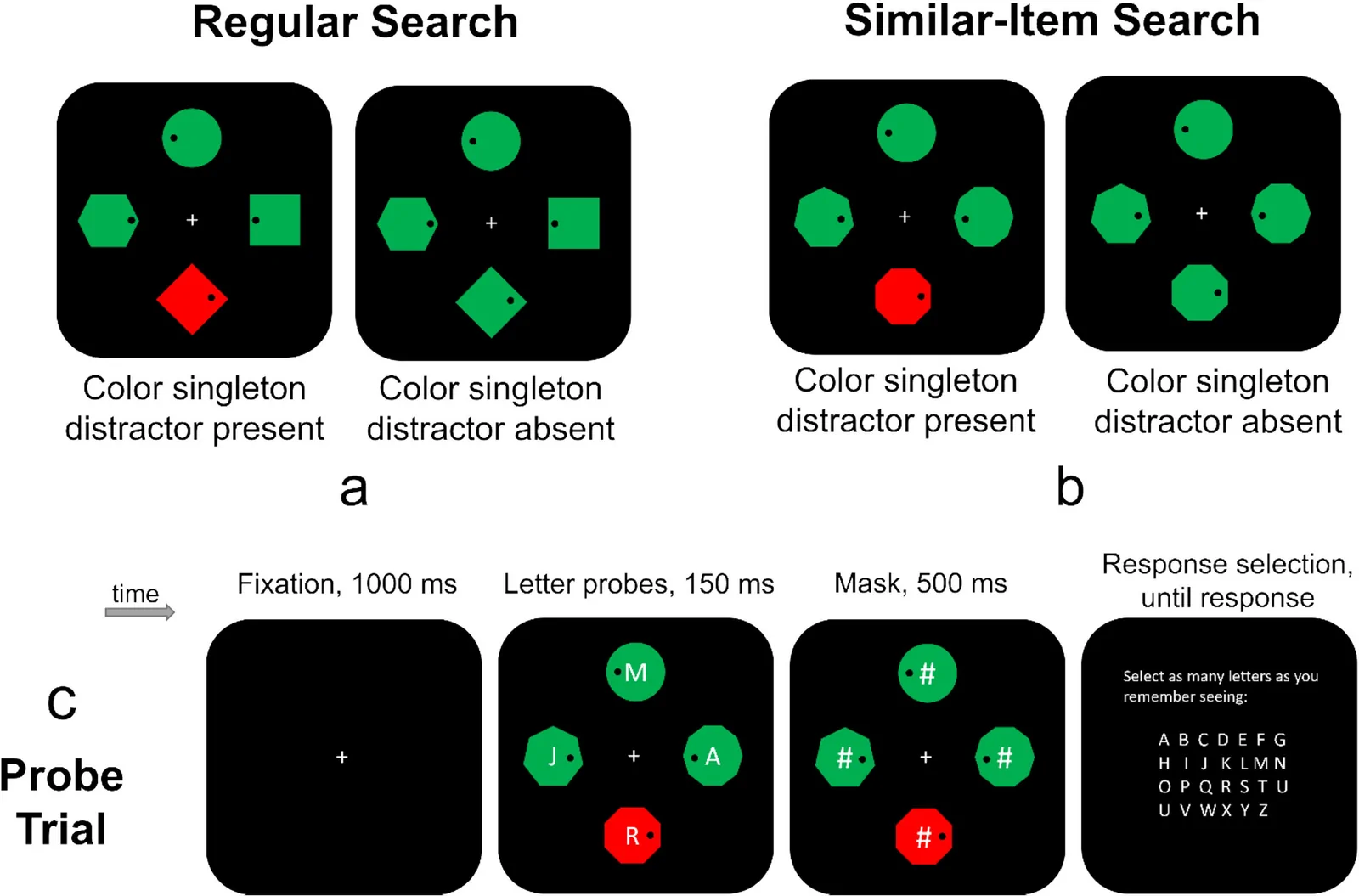
Examples of the regular search task and the similar-item search task from Experiment 1. In both tasks, participants searched for a circle target and reported the location of the contained dot (left or right). The search array items were a fixed color (red or green) for each participant, whereas the distractor, when present, was the alternate color. **a** Examples of search arrays in the regular search task. **b** Examples of search arrays in the similar-item search task. **c** Sequence of events on the probe trials, which were interspersed amongst search trials. The task on probe trials was to identify as many letters as possible. The example shows a trial with a color singleton distractor; other trials had no color singleton. (Color figure online)

Experiment 1 aimed to replicate the basic results of Ruthruff et al. (2020) and Kerzel and Huynh Cong (2024), and manipulated target discriminability in a similar manner by varying the geometric resemblance of the target to the other elements of the search array. In the classical visual search paradigm used to study distractor avoidance (e.g., Gaspelin et al., 2015), distinct shapes (e.g., a square, a diamond, a hexagon, a circle) are used, with the target designated to be one of the shapes, as shown in Fig. 1a (we refer to this as a *regular search*). The target is easily recognizable, which may obviate the need for a highly serial search process. In addition to regular search trials, in the present experiment we also included search arrays in which the target was highly similar to the distractors. As shown in Fig. 1b, on these searches the target was a circle, and the nontarget items were high-sided polygons that are relatively difficult to discriminate from the target circle. We refer to the task of looking for a circle among similar polygons as a *similar-item search*, and we presume that such a search requires a narrow attentional focus. In addition to search trials, letter probe trials were included, similar to the probe method that has been used by others (e.g., Gaspelin et al., 2015; Ma & Abrams, 2023c; Nair et al., 2026). On the probe trials, as shown in Fig. 1c, letters were briefly superimposed onto the shapes of the search array. On these trials, participants were instructed to abandon the search and instead report all of the letters that they saw. Effective distractor avoidance would be indexed by a singleton presence benefit in search-task RT (as shown in Gaspelin et al., 2015; Ma & Abrams, 2023a, b, c), as well as a reduced probe letter report rate for the color singleton distractor compared with that of other nonsalient shapes.

In the present experiment, distractor avoidance and its magnitude was evaluated after confirming that the seriality manipulation was successful. We first assessed search seriality using two performance indicators. First, as search becomes more serial, RTs should increase. Second, increased search seriality should also reduce the probability of reporting probes on the target shape: reduced preattentive guidance should make the initial allocation of attention more likely to miss the target and land on a distractor, especially those less discriminable from the target. Later, in a control experiment (Experiment 4), we more rigorously verified that the similar-item search was more serial than the regular search by examining RT slopes across set sizes. The attentional window account predicts that increased search seriality would benefit distractor avoidance, yielding a larger singleton-presence RT benefit and/or greater probe report disadvantage for singleton distractors in the similar-item search compared with the regular search. On the contrary, if distractor avoidance reflects top-down control, it should not vary with search seriality.

### Method

#### Participants

The initial sample included 24 undergraduate participants (eight men, 16 women), based on a power analysis of the distractor avoidance main effect: Experiments similar in methodology to the regular search task in the present experiment yielded a *d*_z_ = .78 for the effect of singleton presence on RT (Gaspelin et al., 2015, Experiments 2–4). A sample size of 20 would be sufficient to detect such an effect with power = .9 according to G*Power (Erdfelder et al., 1996). In the probe task, the critical comparison involves the probe letter report rates for the color singleton distractor compared with the nonsingleton shapes. Based on an effect size *d*_z_ = .75 from three similar experiments in Gaspelin et al. (2015; reported in Gaspelin & Luck, 2018b), a sample size of 21 is needed to achieve a power of 0.9.

However, after initial data collection we were concerned that the sample appropriate to detect a main effect may not provide enough power to detect the important interaction between distractor avoidance and search seriality, which is typically smaller than the main effect (Aguinis et al., 2005). With the interaction effect unknown, a common heuristic is to estimate its effect size as one-third to one-half the size of the known main effect (Baranger et al., 2023). Given the distractor avoidance effect size of *d*_z_ = 0.78 in search tasks or *d*_z_ = 0.75 in probe tasks (Gaspelin et al., 2015, Experiments 2–4), the discounted estimate of an interaction effect is *f* = 0.18–0.26 (converted from *d*_z_ = 0.25–0.38). This level of Cohen’s *f*-value is also interpreted as a small effect according to convention, representing the smallest meaningful interaction magnitude. Accordingly, the projected sample size was 28–56. To fall within this range, we doubled our previous sample size to *N* = 48. In addition to the initial sample, another 28 undergraduate students were recruited, accounting for attrition and the exclusion of outliers (see details of subject exclusion in the Results). All participants had normal visual acuity and color vision. Informed consent was obtained from each individual.

Because of the post hoc decision to increase sample size, we report both conventional *p*-values and augmented *p*-values following the Sagarin et al. (2014) approach. Importantly, augmented *p*-values are not adjusted *p*-values for individual statistical tests; rather, they estimate the effective Type I error rate associated with the data-dependent sampling procedure itself (i.e., the conditional decision to collect additional participants after inspecting the initial results). Thus, conventional *p*-values reflect the strength of evidence against the null hypothesis for a given analysis, whereas augmented *p*-values reflect how much the flexible sampling procedure may have inflated the probability of obtaining a significant result under the null hypothesis. Augmented *p*-values are reported as a range because the exact stopping rule cannot be known retrospectively. Values close to .05 indicate minimal inflation of the false-positive rate, whereas larger values indicate greater inflation due to the sampling procedure. For example, a result of *p* < .001 with *p*_augmented_ = [.050, .050] indicates that the optional sample augmentation introduced essentially no detectable inflation beyond the nominal 5% false-positive rate, whereas a result of *p* = .04 with *p*_augmented_ = [.090,.120] would indicate that the flexible sampling procedure substantially increased the effective false-positive rate relative to the nominal .05 criterion.

#### Apparatus and stimuli

The experiment was presented on an LCD monitor with a black background at a viewing distance of approximately 50 cm. As shown in Fig. 1a and b, the search array consisted of four shapes, displayed 2° above, below, to the left, and to the right of center. In the regular search task (as shown in Fig. 1a), each array contained a circle (1.4° × 1.4°), a square (1.2° × 1.2°), a diamond (1.6° × 1.6°), and a hexagon (1.5° × 1.5°). In the similar-item search task (as shown in Fig. 1b), each array contained one circle (1.4° × 1.4°) and three high-sided polygons: a heptagon (7 sides; 1.5° × 1.5°), an octagon (8 sides; 1.48° × 1.48°), and an enneagon (9 sides; 1.45° × 1.45°). A black dot (0.2° diameter) was superimposed on either the left or right side of each shape (0.4° from center). In both tasks, items in the search array appeared consistently in either red or green for different participants, with the color singleton distractor, when present, appearing in the alternate color, as shown in Fig. 1a and b. On the probe trials, as shown in Fig. 1c, a white letter (1.2° in height) was superimposed on each search array shape, which later changed into a white pound sign (1.2° in height). A clickable alphabet displaying the 26 English letters (in white; each 1.2° in height) was displayed to collect probe report responses. A fixation cross (0.7° in height) appeared at the center of the display throughout each trial.

#### Procedure

Each participant completed both the regular search and the similar-item search. In both tasks, frequent search trials (two-thirds of the trials) were interleaved with infrequent probe trials (one-third of the trials). Each search trial began with a fixation cross for 1,000 ms, followed by the presentation of the search array until the participant made a response or 2,000 ms had elapsed. The instructions were the same in both tasks: to find the circle and report the location of the dot inside it (left or right). Participants responded by pressing either the left or right arrow key on a computer keyboard. An incorrect or absent response was followed by an error message of “Incorrect!” or “Too slow!”. The next trial began after a 1,000-ms blank screen. On probe trials, after a fixation display of 1,000 ms, a search array (generated based on the same rules as on the search trials) was presented, except that a white letter was superimposed on each shape. On these trials, participants were to try to identify as many letters as possible regardless of what shapes they were on. The letters were visible for 150 ms, and then were each replaced by a pound sign mask for 500 ms. After that, all stimuli disappeared, and an alphabet response collection screen was displayed. Participants, using a mouse, were instructed to click on as many letters as they remembered seeing, in an unspeeded manner. A letter turned into blue upon selection. Feedback for incorrect or missing responses was only provided following a search trial; no feedback was given after a probe trial.

#### Design

In both the regular search and the similar-item search, on one-half of the trials, all items in the array were consistently presented in the same color for each participant, either red or green (*color singleton absent* condition); on another one-half of the trials, one randomly selected noncircle shape was presented in the alternate color (*color singleton present* condition). Participants were explicitly told that the target circle was always among the homogeneously colored shapes, and never uniquely colored, and thus they should ignore the color singleton. The location of the dot on the target circle was equally often on the left or right, and was fully crossed with color singleton presence and color. The locations of the dots on the other shapes were pseudo-randomly selected to ensure that on each trial half of the shapes contained a dot on their left and half of the shapes contained a dot on their right. The four letters on every probe trial were randomly selected without replacement from the 26 letters in the alphabet. The locations of all shapes were randomly selected on each trial.

Within the regular search and the similar-item search tasks, the search and the probe trials were presented in random order. A color singleton distractor was present on one-half of the search trials and one-half of the probe trials. The order of the regular search and the similar-item search as well as the color (red or green) of the search array and the singleton distractor were counterbalanced independently between participants. For our initial sample (*N* = 24), the two tasks were completed in two lab visits, separated by 1–7 days. For the additional sample (*N* = 28), the two tasks were completed in succession in one session. Our pilot results showed that task performance was not affected by whether sessions were separated. For each of the two tasks, before the main experiment, participants completed a practice block of 24 search trials, followed by another practice block of 30 interleaved search and probe trials. For our initial sample, the main experiment for each of the two tasks consisted of three blocks of 108 trials, each including 72 search trials and 36 probe trials. Trial number was reduced slightly in the single-session experiment: three blocks of 72 trials, each including 44 search trials and 24 probe trials.

#### Statistical analysis

In this study, both the traditional null hypothesis significance testing (NHST) and Bayesian analyses were conducted for all results. The inclusion of Bayesian statistics is motivated by the importance of evaluating evidence for null effects, as null effects of target–distractor discriminability on search efficiency are anticipated if the attentional window account is unsupported. In that case, it is central to the study’s aims to acknowledge the true absence of these effects. Bayesian analyses provide additional insight by quantifying evidence in favor of the null hypothesis, complementing traditional NHST, which focuses on rejecting the null. However, to facilitate the dissemination of our findings to a broader audience, we nevertheless included the more common NHST statistics. Importantly, in the present study, the conclusions from NHST and Bayesian analyses were consistent for all results: The significant results identified by NHST also provided support for the alternative hypothesis in the Bayesian analysis, while the null results from NHST correspondingly supported the null hypothesis in the Bayesian framework, with the exception of two non-critical effects noted in footnotes. For convenience, we have chosen to base all primary conclusions and interpretations on NHST results. Bayesian statistics are reported to offer further context, particularly for understanding null results. Our reporting of statistics from both methods but restricting the interpretations to one method follows the suggestions in Dienes (2024).

All Bayesian analyses in this study were conducted using weakly informative priors, as implemented in JASP with its default parameters (JASP Team, 2020). For Bayesian analyses of variance (ANOVAs), a Cauchy distribution with a scale parameter of 0.5 was used as the default prior for effect sizes. For Bayesian *t* tests, a Cauchy prior with a scale of 0.707 was applied to the effect sizes. These priors provide moderate regularization, reflecting minimal prior assumptions about the size of the effects. Bayes factors (BF_10_) were used to quantify evidence in favor of the alternative model, with higher values indicating stronger support for the model with the said effect compared with the null model. Our reporting of the Bayesian statistics follows the guidelines in Depaoli et al. (2020) and Kruschke (2021). BF_10_ values between 1 and 3 are interpreted as providing anecdotal evidence in favor of the alternative hypothesis, BF_10_ values between 3 and 10 are interpreted as moderate evidence, BF_10_ values from 10 to 30 are interpreted as strong evidence, BF_10_ values between 30 and 100 are interpreted as very strong evidence, and BF_10_ values greater than 100 are interpreted as extreme evidence. Conversely, the inverse of these ranges is used to interpret evidence in favor of the null hypothesis.

#### Transparency and openness

We report how we determined our sample size, all data exclusions, all manipulations, and all measures in the study, and the study follows JARS (Appelbaum et al., 2018). Trial-level data and analysis code for all experiments has been made publicly available online (https://osf.io/njvu2). Data were analyzed using R (Version 4.0.0; R Core Team, 2020) and the Bayesian analyses were conducted in JASP (JASP Team, 2020). This study was not preregistered.

### Results

In the total sample, two participants withdrew from the experiment after the first lab visit, and their incomplete data were not included in the analysis. Other participants had to meet an overall accuracy criterion of 80% or greater in both the regular search and the similar-item search, and also to correctly report an average of 0.8 letters or more per trial in the probe tasks to be included in the analysis. Two participants were removed due to low probe report accuracy, leaving the remaining 48 participants included in the analyses. For each individual, search trials with incorrect or missing responses (regular search: 2.9% of total trials; similar-item search: 3.1% of total trials) or RTs beyond 2SD of the mean of each cell condition were not included in RT analysis (regular search: 4.2% of total trials; similar-item search: 3.9% of total trials).

#### Search-task analysis

**Fig. 2:**
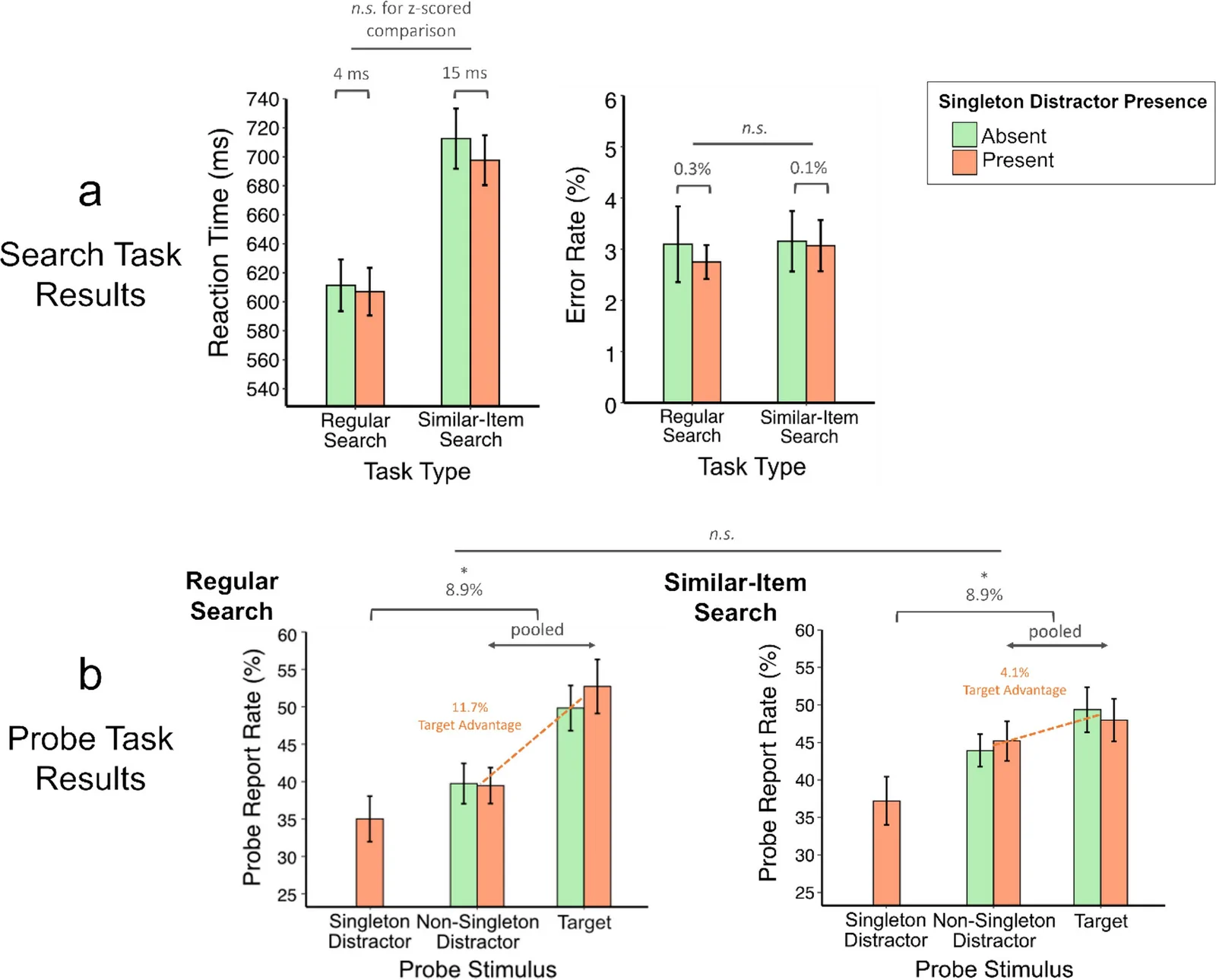
The regular search-task and the similar-item search-task results from Experiment 1. **a** Search-task RT (left panel) and error rate (right panel) as a function of task type and color singleton distractor presence. **b** Results from the probe trials in the regular search (left panel) and the similar-item search (right panel). The probe report rate was lower for the color singleton distractor than for the pooled target and nonsingleton shapes in both tasks, suggesting effective distractor avoidance. The size of the avoidance effect did not differ across tasks. Error bars represent within-subject standard errors (Cousineau et al., 2021). (Color figure online)

The search-task results are shown in Figure 2a. To check the effectiveness of the target–distractor similarity manipulation, performance in the two tasks was compared against each other using paired-samples *t* tests. RTs were significantly slower in the similar-item search (705 ms) compared with the regular search (609 ms), *t*(47) = 12.30, *p* < .001, *d* = 1.02, *p*_augmented_ = [.050, .050], BF_10_ > 100. This validates participants’ sensitivity to the increased physical resemblance in the similar-item search. The difference in error rate between the similar-item search (2.9%) and the regular search (3.1%) was not significant, *t*(47) = 0.69, *p* = .491, *d* = 0.07, *p*_augmented_ = [.394, .498], BF_10_ = 0.20.

To facilitate cross-task comparison, RTs from the similar-item search and the regular search were converted to *Z* scores. A 2 (task type: similar-item search, regular search) × 2 (color singleton distractor presence: present, absent) repeated-measures ANOVA was conducted separately on RT and error rate. The main effect of singleton distractor presence was significant, *F*(1, 47) = 7.40, *p* = .009, η_p_^2^ = 0.14, *p*_augmented_ = [.053, .055], BF_10_ = 2.34. Overall, RTs were faster when a color singleton distractor was present (652 ms) compared with when it was absent (662 ms)—a singleton presence benefit suggesting avoidance of the distractor. As expected with the RTs converted to *Z* scores, the main effect of task type was not significant, *p* = 1.000. The interaction between singleton distractor presence and task type was not significant, *F*(1, 47) = 2.28, *p* = .138, η_p_^2^ = .05, *p*_augmented_ = [.078, .161], BF_10_ = 0.31, suggesting a comparable magnitude of distractor avoidance in the two tasks.

The analysis of error rates yielded no differences. The main effect of color singleton distractor presence, the main effect of task type, and the interaction between them were all not significant, *F*(1, 47) = 0.48, *p* = .491, η_p_^2^ = 0.01, *p*_augmented_ = [.302, .498], BF_10_ = 0.15; *F*(1, 47) = 0.62, *p* = .434, η_p_^2^ = 0.01, *p*_augmented_ = [.356, .442], BF_10_ = 0.16; *F*(1, 47) = 0.19, *p* = .668, η_p_^2^ = 0.00, *p*_augmented_ = [.480, .671], BF_10_ = 0.03, respectively.

#### Probe-task analysis

The probe-task results are shown in Fig. 2b. On average participants reported 1.82 letters per trial in the regular search and 1.91 letters per trial in the similar-item search, 92.0% and 93.5% of which were actually present in the display, respectively, in the two tasks.

Previous studies using the regular search display showed that the letter probes on the target shape were reported at a higher rate than those on the nonsingleton, nontarget shapes (e.g., Gaspelin et al., 2015). If target–distractor discriminability was effectively reduced in the similar-item search, the target shape should be harder to locate during the brief exposure of the probe letters, and the advantage of target probe reporting should thus be less pronounced in the similar-item search. To check that, a 2 (task type: regular search, similar-item search) × 2 (probe stimulus: target, nonsingleton shape) × 2 (color singleton distractor presence: present, absent) repeated-measures ANOVA was conducted on probe report rate. The only significant main effect was the main effect of probe stimulus, *F*(1, 47) = 50.56, *p* < .001, η_p_^2^ = 0.52, *p*_augmented_ = [.050, .050], BF_10_ > 100. Overall, the letters on the target were reported at a higher rate than those on the nonsingleton shapes, confirming the effectiveness of the probe method in measuring attention to individual search array elements. The only significant interaction was the interaction between probe stimulus and task type, *F*(1, 47) = 15.09, *p* < .001, η_p_^2^ = 0.24, *p*_augmented_ = [.050, .050], BF_10_ = 51.73. The advantage in reporting the letters on the target compared with the nonsingleton shapes was greater in the regular search (11.7% target advantage) than in the similar-item search (4.1% target advantage). This verifies that the target was more difficult to identify in the similar-item search, confirming the effectiveness of the manipulation. None of the other main effects or interactions were significant.

To examine the avoidance of the color-singleton distractor, past studies typically use the nonsingleton probe report rates as a baseline to compared against singleton probe report rate (e.g., Gaspelin et al., 2015). However, in the present study, as the target shape became less discriminable and harder to locate in the similar-item search, initial attention appeared to have consequently selected the nonsingleton shapes at a higher rate, possibly inflating the difference between the nonsingleton probe report rate and that of the singleton distractor—thereby overestimating the magnitude of distractor avoidance. To account for this influence, we calculated an average probe report rate including the two nonsingleton shapes and the target, on singleton distractor present trials. This pooled probe report rate serves as an adjusted baseline reflecting the amount of attention allocated to target-colored shapes overall, and was used to compare against the probe report rate of the singleton. A 2 (task type: regular search, similar-item search) × 2 (probe stimulus: color singleton distractor, target-colored shapes pooled) repeated-measures ANOVA was conducted on probe report rate on singleton distractor-present trials. The main effect of probe stimulus was significant, *F*(1, 47) = 14.21, *p* < .001, η_p_^2^ = 0.23, *p*_augmented_ = [.050, .050], BF_10_ = 44.99. The singleton probe (36.1%) had a much lower report rate than the pooled baseline (45.0%). The main effect of task type was also significant, *F*(1, 47) = 4.15, *p* = .047, η_p_^2^ = 0.08, *p*_augmented_ = [.072, .083], BF_10_ = 0.84,^1^ with slightly higher overall probe report rates in the similar-item search than regular search. The interaction between task type and probe stimulus was not significant, *F*(1, 47) = 0.00, *p* = .983, η_p_^2^ = 0.00, *p*_augmented_ = [.966, .983], BF_10_ = 0.22, respectively. Probes on the salient distractor were reported at a similar level of reduced rates compared with the pooled baseline in regular search (8.9% below baseline) and similar-item search (also 8.9% below baseline). The serial search manipulation was shown to have no effect on distractor avoidance in the probe task.

### Discussion

The reduced target discriminability manipulation in the similar-item search was confirmed to be effective based on the results of both the search and the probe tasks. Despite that, the probe task results suggest that attentional allocation to a salient distractor could be avoided, and the size of the avoidance effect did not differ as a function of target–distractor discriminability. That is, contrary to the prediction of the attentional window account, increasing the seriality of the search failed to boost distractor ignoring. Instead, our results are more consistent with the explanation that top-down control can guide attention to avoid irrelevant distractors regardless of search seriality.

## Experiment 2

**Fig. 3:**
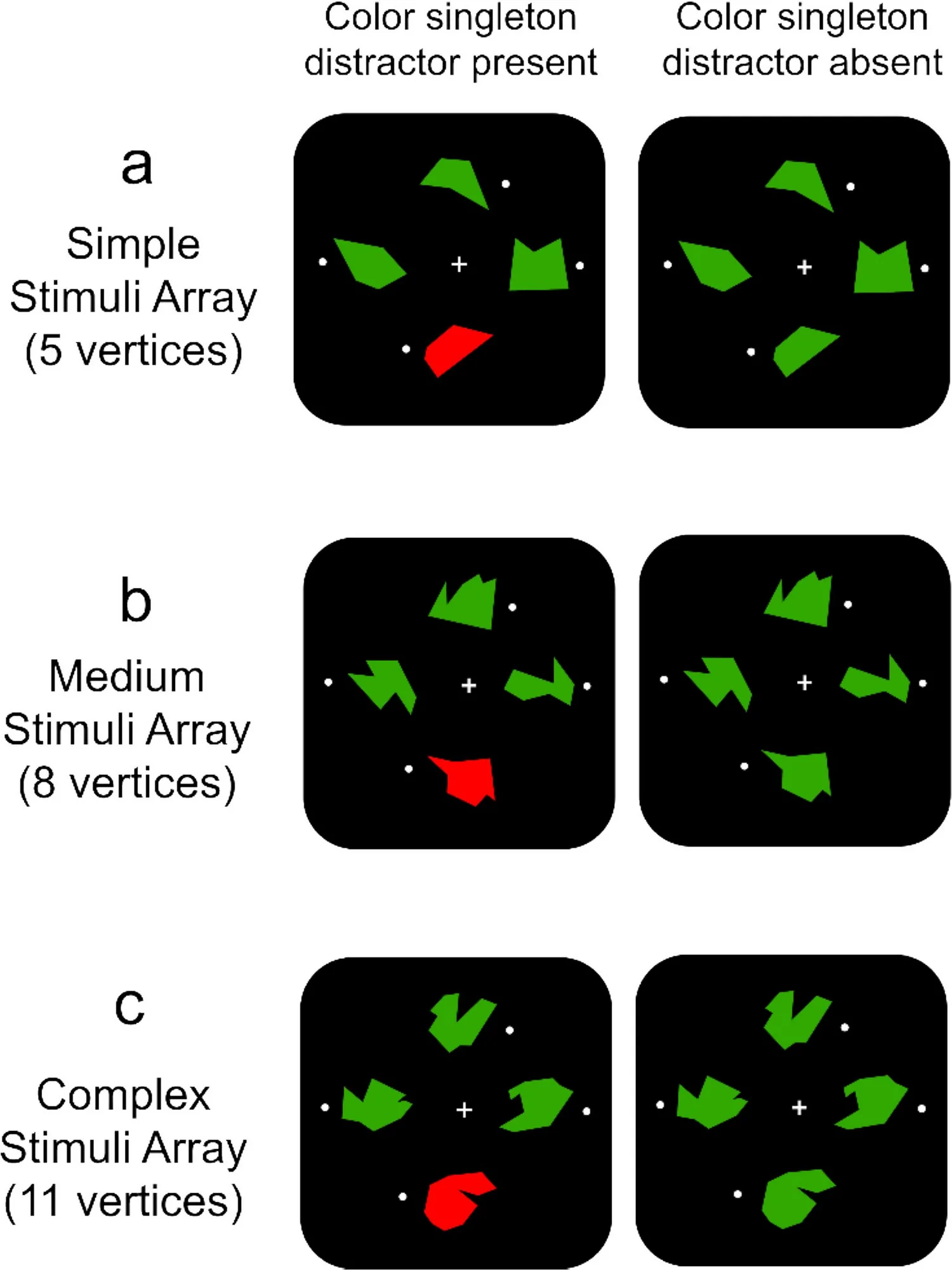
Search tasks with varied stimulus complexity from Experiment 2. **a** Examples of search arrays consisting of simple stimuli, each with five vertices. **b** Examples of search arrays consisting of medium stimuli, each with eight vertices. **c** Examples of search arrays consisting of complex stimuli, each with 11 vertices. In each of the three complexity conditions, participants searched for a predefined target shape matched in complexity with the distractors in the array, and reported the location of a dot (left or right) adjacent to the target shape. Letter probe trials (not shown in the figure) were randomly interspersed amongst the search trials. (Color figure online)

An alternative way to reduce target discriminability is to increase the mental representational difficulty of the search stimuli by making their visual patterns more complex. Perceived distinctiveness of individual visual stimuli within an array was found to decrease as the pattern complexity of the stimuli increased (Sun & Firestone, 2021). For example, Sun and Firestone (2021) created a range of abstract shape stimuli varying in complexity–defined on the basis of the number of sides and the angles of their inner skeletal structures. In a series of visual search conditions with stimuli at different levels of complexity, a monotonic relationship was found between stimulus complexity and search performance: Target detection was the least efficient among an array of highly complex stimuli, suggesting their low perceptual distinctiveness. Using the same method as in Sun and Firestone (2021), the present experiment manipulated the target–distractor discriminability by varying their pattern complexities. Each participant performed a series of visual search tasks among items of three complexity levels, as shown in Fig. 3. In each complexity condition, participants searched for a predefined target shape that had matching complexity with the distractor shapes in the array. As in Experiment 1, the search array was a fixed color (either red or green) for each participant, with the color singleton distractor the alternate color. Probe trials were interleaved with the search trials. The attentional window account predicts that a more serial search would facilitate distractor avoidance, and thus the singleton presence benefit in search RT and/or the disadvantage in probe reports for the singleton distractor would increase as stimulus complexity increases. On the contrary, the top-down control accounts predict that the salient distractor should be avoided to a similar extent regardless of search seriality.

### Method

#### Participants

The sample size for Experiment 2 was determined based on the same criteria as the initial sample of Experiment 1. A new set of 24 undergraduate students (eight men, 16 women) were recruited. All participants had normal visual acuity and color vision. Informed consent was obtained from each individual.

#### Stimuli

Abstract shape stimuli were generated using the methods in Sun and Firestone (2021). The number of sides for each shape was predefined to be 5, 8, and 11 separately for the simple, medium, and complex conditions, and was the same for all stimuli within each complexity level. A set of four shapes was generated for each complexity level, creating a total of 12 distinct shapes, which are illustrated in Fig. 3. In addition to the number of vertices, several other rules were used in shape generation, which include that all inner and outer angles of the shapes had to fall between 20° and 160°, and the minimum length of the vertices had to be at least 1% of the width of the shape. Stimulus complexity manipulated with this method has been shown to effectively influence perceptual processing in several experiments (Sun & Firestone, 2021).

Similar to Experiment 1, the search array consisted of four shapes, displayed 4° above, below, to the left, and to the right of center. Each shape was approximately 4° × 3° in size. One of the four shapes in each condition was selected to be the search target. A white dot (0.2° diameter) appeared either to the left or right of each shape (0.4° from the boundary). Items in the search array appeared in either red or green for each participant, with the color singleton distractor, when present, appearing in the alternate color, as shown in Fig. 3. On probe trials, the probe letters were 1.6° in height. A fixation cross (0.7° in height) appeared at the center of the display throughout each trial.

#### Procedure

The procedure was identical to that of Experiment 1, except that a different target shape was specified for each complexity condition.

#### Design

The same rules as in Experiment 1 were used to determine the stimulus color, stimulus location, dot location, color singleton presence (with a probability of 50%), and their relation on each search trial. Also, the same rules as in Experiment 1 were used to generate the probe letters on the probe trials. Each participant completed the experiment under the three different stimulus complexity levels in two different lab visits. One complexity level was tested in the first lab visit, and the two remaining levels were tested in the second visit. The order of the three complexity levels and the color assignments for the search array elements and the singleton distractor were counterbalanced between participants. In each complexity condition, one of the four shapes was randomly selected for each participant to be the search target. For each of the three tasks, before the main experiment, participants completed a practice block of 24 search trials, followed by another practice block of 24 interleaved search and probe trials; The main experiment for each complexity level consisted of 2 blocks of 108 trials, each including 72 search trials and 36 probe trials.

### Results

Three participants withdrew from the experiment after the first lab visit, and their incomplete data were not included in the analysis. Other participants had to meet an overall accuracy criterion of 80% or greater in all three search tasks, and also to correctly report an average of 0.8 letters or greater per trial in the probe tasks to be included in the analysis. Of the remaining 21 participants, one person was removed due to low probe report accuracy, leaving 20 participants included in the analysis. For each individual, trials with incorrect or missing responses (simple: 2.4% of total trials; medium: 2.8% of total trials; complex: 3.5% of total trials) or RTs beyond 2 standard deviations of the mean of each cell condition were not included in RT analysis (simple: 4.7% of total trials; medium: 4.2% of total trials; complex: 3.5% of total trials).

#### Search-task analysis

**Fig. 4:**
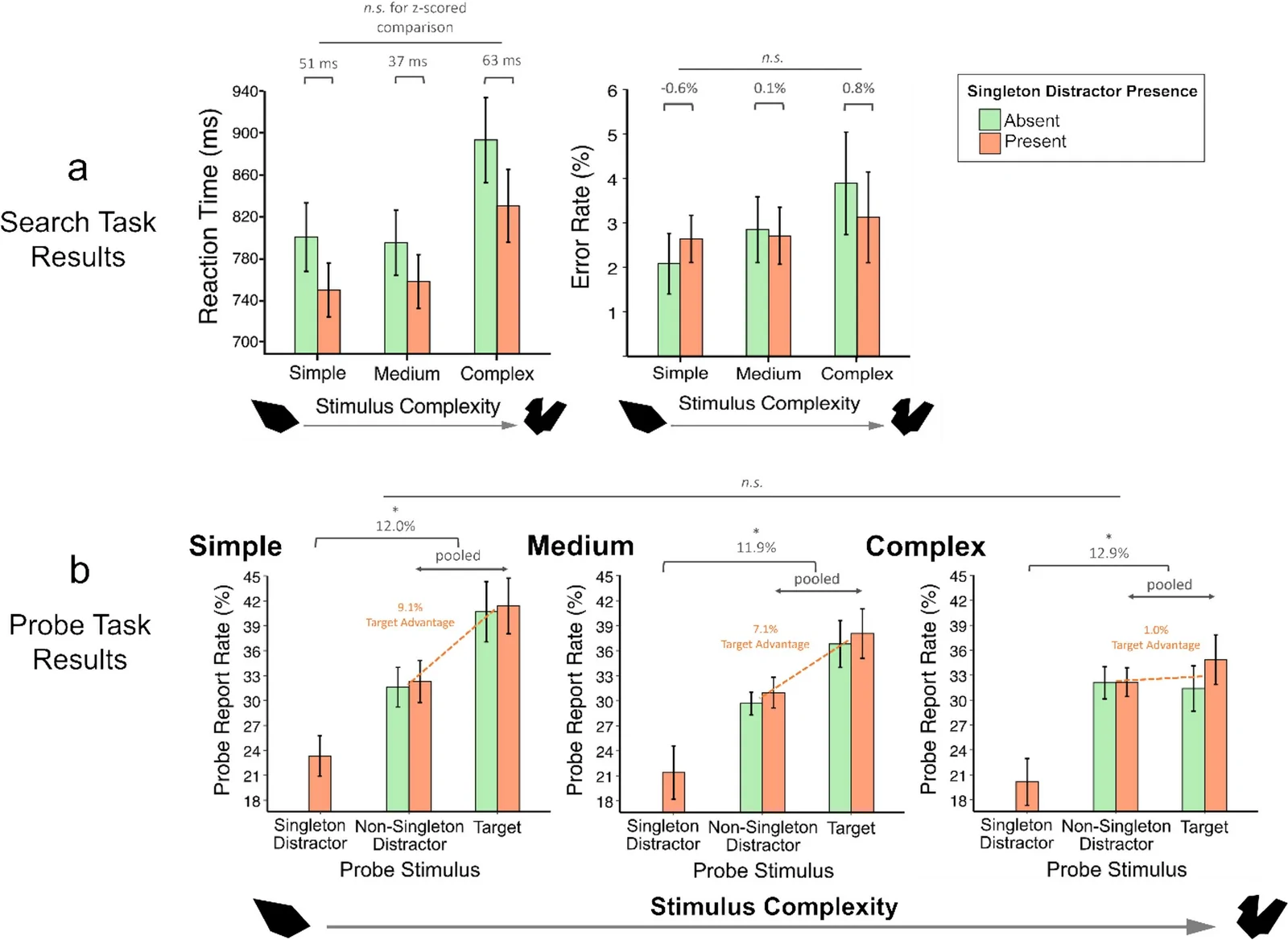
Results of the search and the probe tasks from Experiment 2. **a** Search-task RT (left panel) and error rate (right panel) as a function of stimulus complexity and color singleton distractor presence. The presence of a color-singleton distractor led to performance benefits at all three complexity levels. **b** Results from the probe trials in the simple (left panel), medium (middle panel), and complex (right panel) conditions. The probe report rate was lower for the color singleton distractor than for the nonsingleton shapes at all three complexity levels, suggesting effective distractor avoidance. The size of the avoidance effect did not differ across complexity levels. Error bars represent within-subject standard errors (Cousineau et al., 2021). (Color figure online)

The results are shown in Fig. 4a. To check whether the search efficiency effectively decreased as a function of stimulus complexity, performance in the three complexity conditions was compared in one-way repeated-measures ANOVAs. RT significantly increased as stimulus complexity increased, *F*(2, 38) = 4.69, *p* = .015, η_p_^2^ = 0.20, BF_10_ = 3.62. The simple level had the fastest RT (775 ms), followed by the medium level (776 ms), and the complex level (862 ms). Post hoc *t* tests with Bonferroni correction showed that the simple condition was significantly faster than the complex condition, *t*(19) = 2.67, *p*_bonferroni_ = .033, *d* = 0.61, BF_10_ = 5.10. The medium condition was also significantly faster than the complex condition, *t*(19) = 2.63, *p*_bonferroni_ = .037, *d* = 0.60, BF_10_ = 1.56. The difference between the simple and medium conditions was not significant, *t*(19) = 0.04, *p*_bonferroni_ = 1.000, *d* = 0.01, BF_10_ = 0.23. These results confirm participants’ sensitivity to the increased target–distractor similarity from the simple to the complex task. The error rates of the three conditions (simple: 2.4%; medium: 2.8%; complex: 3.5%) did not significantly differ from each other, *F*(2, 38) = 1.58, *p* = .219, η_p_^2^ = 0.08, BF_10_ = 0.41.

To facilitate cross-condition comparison, RTs within each complexity level were converted to *Z* scores. A 3 (complexity level: simple, medium, complex) × 2 (color singleton distractor presence: present, absent) repeated-measures ANOVA was conducted separately on RT and error rate. The main effect of color singleton distractor presence was significant, *F*(1, 19) = 32.93, *p* < .001, η_p_^2^ = 0.63, BF_10_ > 100. Overall, the presence of a color singleton distractor speeded up the search in the three tasks. The main effect of complexity was not significant as expected with the RTs of each task converted to *Z* scores, *p* = 1.000. The interaction between distractor presence and complexity was not significant, *F*(2, 38) = 0.52, *p* = .601, η_p_^2^ = 0.03, BF_10_ = 0.23. In all three complexity conditions, the presence of a color singleton distractor led to a similar level of performance benefit.

The analysis of error rates revealed no differences. The main effect of color singleton distractor presence, the main effect of complexity, and the interaction between them were all not significant, *F*(1, 19) = 0.10, *p* = .758, η_p_^2^ = 0.01, BF_10_ = 0.27; *F*(2, 38) = 1.58, *p* = .219, η_p_^2^ = 0.08, BF_10_ = 0.48; *F*(2, 38) = 1.07, *p* = .353, η_p_^2^ = 0.05, BF_10_ = 0.31, respectively.

#### Probe-task analysis

The probe-task results are shown in Figure 4b. On average participants reported 1.57 letters per trial in the simple condition, 1.44 letters per trial in the medium condition, and 1.47 letters per trial in the complex condition, 84.5%, 85.8%, and 84.1% of which were actually present in the display, respectively, in the three tasks.

If the target discriminability was effectively reduced as stimulus complexity increased, participants should be less likely to locate the target with a brief exposure to the display in the complex condition compared with the simpler conditions. To check that, a 3 (complexity level: simple, medium, complex) × 2 (probe stimulus: target, nonsingleton shape) × 2 (color singleton distractor presence: present, absent) repeated-measures ANOVA was conducted on probe report rate. The only significant main effect was the main effect of probe stimulus, *F*(1, 19) = 13.93, *p* = .001, η_p_^2^ = 0.42, BF_10_ = 2.75. Overall, the letters on the target were reported at a higher rate than those on the nonsingleton shapes, confirming the effectiveness of the probe method in measuring attention to individual search array elements. The only significant interaction was the interaction between probe stimulus and complexity, *F*(2, 38) = 4.10, *p* = .025, η_p_^2^ = 0.18, BF_10_ = 3.14. The advantage in reporting the letters on the target compared with the nonsingleton shapes decreased as stimulus complexity increased: simple (9.1% target advantage), medium (7.1% target advantage), complex (1.0% target advantage). Post hoc *t* tests with Bonferroni corrections showed that the probe report rate was significantly higher for the target than for the nonsingleton shapes in the simple condition and the medium condition, *t*(19) = 3.97, *p*_bonferroni_ = .003, *d* = 0.78, BF_10_ = 7.44; *t*(19) = 3.10, *p*_bonferroni_ = .046, *d* = 0.61, BF_10_ = 19.71, respectively. However, this target reporting advantage was not significant in the complex condition, *t*(19) = 0.44, *p*_bonferroni_ = 1.000, *d* = 0.09, BF_10_ = 0.27. This confirms the effectiveness of the complexity manipulation in decreasing target discriminability. None of the other main effects or interactions were significant.

As the target shape became less discriminable in the more complex conditions, initial attention was more likely to miss the target but land on other target-like nonsingleton distractors, inflating the nonsingleton baseline against which distractor avoidance was typically evaluated. To avoid such biases, as in Experiment 1, we again adjusted the baseline level of attention by calculating a pooled probe report rate including the two nonsingleton shapes and the target on singleton distractor present trials. A 3 (complexity level: simple, medium, complex) × 2 (probe stimulus: color singleton distractor, target-colored shapes pooled) repeated-measures ANOVA was conducted on probe report rate on singleton distractor present trials. The main effect of probe stimulus was the only significant result, *F*(1, 19) = 21.74, *p* < .001, η_p_^2^ = 0.53, BF_10_ > 100. Overall, the singleton probe (21.6%) was reported at a much lower rate than the pooled baseline (33.9%). The main effect of complexity and the interaction between complexity and probe stimulus were all not significant, *F*(2, 38) = 1.31, *p* = .283, η_p_^2^ = 0.06, BF_10_ = 0.20; *F*(2, 38) = 0.03, *p* = .970, η_p_^2^ = 0.00, BF_10_ = 0.14, respectively. Probes on the salient distractor were reported at a similar level of reduced rates compared with the pooled baseline in in the simple (12.0% below baseline), medium (11.9% below baseline), and complex (12.9% below baseline) conditions. The serial search manipulation was shown to have no effect on distractor avoidance in the probe task.

### Discussion

The results of Experiment 2 supplement those of Experiment 1. The reduced target discriminability manipulation across the three complexity conditions produced the expected effect on the search based on the results from both the search and the probe trials. Despite that, the probe-task results suggest that while attentional allocation to the salient distractor could be avoided, the size of the attentional avoidance effect did not differ across the complexity conditions. That is, inconsistent with the prediction of the attentional window account, increased search seriality failed to boost the magnitude of distractor avoidance. By ruling out this passive exclusion mechanism, our results instead align more with the top-down control mechanism in distractor avoidance.

## Experiment 3

**Fig. 5:**
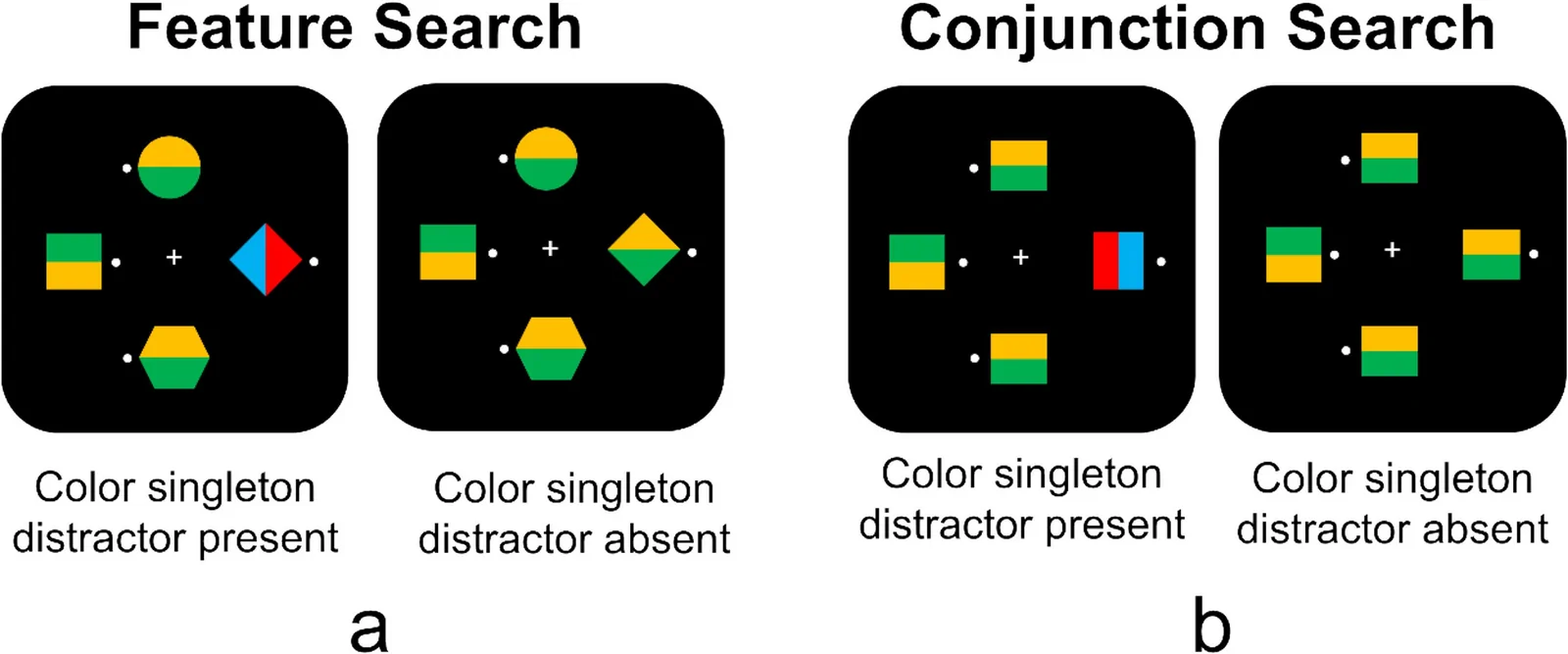
The feature-search and the conjunction-search tasks from Experiment 3. **a** Examples of the feature-search arrays. Items in the search array were either red and blue, or green and yellow (for different participants) with horizontal strips. The color singleton distractor, when present, appeared in the alternate color combination and with vertically oriented color strips. Participants searched for a target square, and reported the location of a dot relative to it (left or right). **b** Examples of the conjunction-search arrays. The elements contained color strips arranged based on the same rules as in the feature search. Participants searched for a target square with the top color strip a specific color (green in the example), and reported the location of a dot relative to it (left or right). (Color figure online)

Experiments 1 and 2 both manipulated bottom-up factors and varied the appearance of the stimuli to create different levels of search seriality. In addition to the influence of bottom-up factors, search seriality can also be affected by top-down influences associated with the demands of the task. In particular, conjunction searches that define the target based on a combination of features tend to reveal reduced efficiency compared with simple searches that define the target with a single feature, even when using the same stimulus arrays (Egeth et al., 1984; Treisman & Gelade, 1980; Zohary & Hochstein, 1989). The binding of multiple features to an object requires focused attention, thus invoking highly serial searches (Treisman & Gelade, 1980). Experiment 3 created a conjunction-search task by having all stimuli in the array possessing the same two colors, but by defining the target by a specific spatial arrangement of the two colors. As shown in Fig. 5b, each search array in the *conjunction-search task* consisted of four bi-color squares. The target square contained the same two colors as the nontargets (either red and blue, or green and yellow), but with the color strips arranged in a distinct prespecified configuration (viz., with the red or green strip on top). Having shared features between the target and distractors is considered to reduce perceived distinctiveness of the target, and to force participants to attend to the relation between the two features to identify the target (Treisman & Gelade, 1980). As in Experiment 1, the search array always appeared in a fixed color pair for each participant, with the color singleton distractor appearing in the alternate color pair and with the color strips oriented vertically. The strength of avoidance of a salient singleton distractor in the conjunction search was measured. As a control condition, the same group of participants also performed a single-feature-search task with distinct shapes, as shown in Fig. 5a, where the search array elements were comprised of color strips arranged based on the same rules as in the conjunction search, except that the search target was defined as a prespecified shape (viz., a square)—a single feature—rather than the relation between two features. As in Experiment 1, probe trials were interleaved with the search trials in both the conjunction search and the feature search. Search seriality was preliminarily assessed in the present experiment via the efficiency in the search task and the advantage in reporting target letters in the probe task. Experiment 4 further validated the seriality manipulation by demonstrating a steeper search slope (as a function of set size) in the conjunction-search task.

### Method

#### Participants

The sample size for Experiment 3 was determined based on the same criteria as in Experiments 1 and 2. A new set of 24 undergraduate students (eight men, 16 women) were recruited. All participants had normal visual acuity and color vision. Informed consent was obtained from each individual.

#### Stimuli

In the conjunction search, as shown in Figure 5b, each search array consisted of four squares (1.5° × 1.5°) each comprised of two color strips (either red and blue, or green and yellow, consistently throughout the experiment for each participant), displayed 3° above, below, to the left, and to the right of center. The target square was defined to have horizontally aligned color strips, with either red or green (for different participants) on top. The nonsingleton distractors possessed the same two colors as the target, but in a vertically flipped arrangement (with either red or green at the bottom). The color singleton distractor, when present, was in the alternate color pair and had color strips vertically aligned^2^ (e.g., a green–yellow color singleton distractor among a red–blue search array, and vice versa). A white dot (0.2° diameter) appeared either to the left or right of each shape (0.3° from the boundary). In the feature search, as shown in Fig. 5a the color combinations were identical to those in the conjunction search, except that the array elements were a square target (1.2° × 1.2°), accompanied by circle (1.4° × 1.4°), diamond (1.6° × 1.6°), and hexagon (1.5° × 1.5°) nontarget shapes. On probe trials, as in Experiment 1, the probe letters were presented in white, 1.2° in height. A fixation cross (0.7° in height) appeared at the center of the display throughout each trial.

#### Procedure

The procedure was identical to that of Experiment 1.

#### Design

The same rules as in Experiment 1 were used to determine the stimulus color, stimulus location, dot location, color singleton presence (with a probability of 50%), and their relation on each search trial. Also, the same rules as in Experiment 1 were used to generate the probe letters on the probe trials. Each participant completed the conjunction search and the feature search in two different lab visits. The order of the two tasks and the color assignments for the search array elements and the singleton distractor were counterbalanced between participants. For each of the two tasks, before the main experiment, participants completed a practice block of 28 search trials, followed by another practice block of 24 interleaved search and probe trials. The main experiment of each of the two tasks consisted of two blocks of 108 trials, each including 72 search trials and 36 probe trials.

### Results

Six participants withdrew from the experiment after the first lab visit, and their incomplete data were not included in the analysis. Other participants had to meet an overall accuracy criterion of 80% or greater in both the conjunction-search and the feature-search tasks, and also to correctly report an average of 0.8 letters or greater per trial in the probe tasks to be included in the analysis. Of the remaining 18 participants, one person was removed due to low probe report accuracy in the feature search, and one person was removed due to low probe report accuracy in both search tasks, leaving 16 participants included in the analysis. Because the sample size after participant removal was lower than the minimum projected sample size to ensure adequate power, we recruited an additional five participants. Among the replacement participants, one person was removed due to low probe report accuracy in both search tasks, leaving a total of 20 participants included in the analysis. For each individual, trials with incorrect or missing responses (conjunction search: 2.7% of total trials; feature search: 1.6% of total trials) or RTs beyond 2 standard deviations of the mean of each cell condition were not included in RT analysis (conjunction search: 4.0% of total trials; feature search: 4.1% of total trials).

#### Search-task analysis

**Fig. 6:**
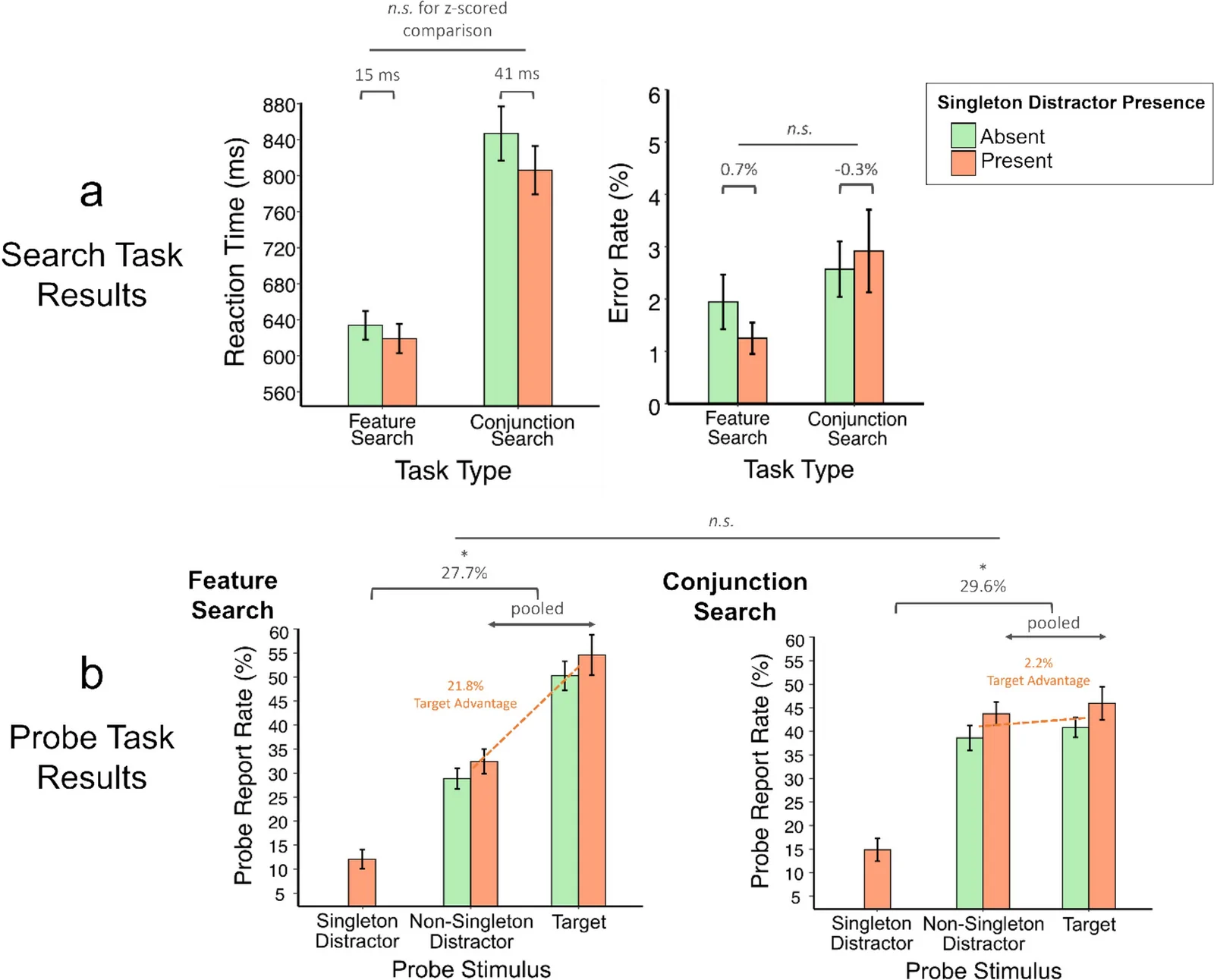
The conjunction-search and the feature-search results from Experiment 3. **a** Results in search-task RT (left panel) and error rate (right panel) as a function of task type and color singleton distractor presence. **b** Results from the probe trials in the feature search (left panel) and the conjunction search (right panel). The probe report rate was lower for the color singleton distractor than for the nonsingleton shapes in both tasks, suggesting effective distractor avoidance. The size of the avoidance effect did not differ across tasks, after adjustment of the baseline reference. Error bars represent within-subject standard errors (Cousineau et al., 2021). (Color figure online)

The results are shown in Fig. 6a. To check whether the search efficiency effectively decreased in the conjunction search compared with that in the feature search, performance in the two tasks was compared against each other with paired-samples *t* tests. RTs were significantly slower in the conjunction search (826 ms) compared with the feature search (626 ms), *t*(19) = 8.78, *p* < .001, *d* = 1.96, BF_10_ > 100. This is consistent with the participants’ search mode being more serial in the conjunction search. Participants also made marginally significantly more errors in the conjunction search (2.8%) than in the feature search (1.6%), *t*(19) = 1.91, *p* = .072, *d* = 0.54, BF_10_ = 1.05.

To facilitate cross-task comparison, RTs from the conjunction search and the feature search were converted to *Z* scores. A 2 (task type: conjunction search, feature search) × 2 (color singleton distractor presence: present, absent) repeated-measures ANOVA was conducted separately on RT and error rate. The main effect of singleton distractor presence was significant, *F*(1, 19) = 30.36, *p* < .001, η_p_^2^ = 0.62, BF_10_ > 100. Overall, RTs were faster when a color singleton distractor was present (713 ms) compared with when it was absent (740 ms)—a singleton presence benefit suggesting avoidance of the distractor. As expected with the RTs converted to *Z* scores, the main effect of task type was not significant, *p* = 1.000. The interaction between singleton distractor presence and task type was also not significant, *F*(1, 19) = 1.45, *p* = .244, η_p_^2^ = 0.07, BF_10_ = 0.34, suggesting an equivalent magnitude of distractor avoidance in the two tasks.

The analysis of error rates produced consistent results. As noted earlier, the main effect of task type was marginally significant, *F*(1, 19) = 3.64, *p* = .072, η_p_^2^ = 0.16, BF_10_ = 1.24, with participants making more errors in the conjunction search than in the feature search. The main effect of color singleton distractor presence and the interaction between them were not significant, *F*(1, 19) = 0.16, *p* = .691, η_p_^2^ = 0.01, BF_10_ = 0.32; *F*(1, 19) = 1.41, *p* = .250, η_p_^2^ = 0.07, BF_10_ = 0.67, respectively.

#### Probe-task analysis

The probe-task results are shown in Figure 6b. On average participants reported 1.80 letters per trial in the conjunction search and 1.59 letters per trial in the feature search, 84.4% and 84.3% of which were actually present in the display, respectively, in the two tasks.

If target–distractor discriminability was effectively reduced in the conjunction search, the target shape should be harder to locate with a brief exposure to the display. The advantage of reporting the probe letters on the target shape should thus be less pronounced in the conjunction search than in the feature search. To check that, a 2 (task type: conjunction search, feature search) × 2 (probe stimulus: target, nonsingleton shape) × 2 (color singleton distractor presence: present, absent) repeated-measures ANOVA was conducted on probe report rate. The main effect of probe stimulus was significant, *F*(1, 19) = 29.32, *p* < .001, η_p_^2^ = 0.61, BF_10_ > 100. Overall, the letters on the target were reported at a higher rate than those on the nonsingleton shapes, confirming the effectiveness of the probe method in measuring attention to individual search array elements. The main effect of distractor presence was also significant, *F*(1, 19) = 30.36, *p* < .001, η_p_^2^ = 0.62, BF_10_ = 92.96. More letters were reported on the distractor present trials compared with the distractor absent trials, consistent with the singleton presence benefit on the search trials. The only significant interaction was the interaction between probe stimulus and task type, *F*(1, 19) = 29.69, *p* < .001, η_p_^2^ = 0.61, BF_10_ > 100. The advantage in reporting the letters on the target compared with the nonsingleton shapes was greater in the feature search (21.8% target advantage) than in the conjunction search (2.2% target advantage). This verifies that the target was more difficult to identify in the conjunction search, confirming the effectiveness of the manipulation to increase seriality. None of the other main effects or interactions were significant.

As the target shape became less discriminable and harder to locate in the more complex conditions, it is possible that initial attention was more likely to miss the target but land on other target-like nonsingleton distractors, inflating the nonsingleton baseline against which distractor avoidance was typically evaluated. To avoid such biases, as in Experiments 1 and 2, we again adjusted the baseline level of attention by calculating a pooled probe report rate including the two nonsingleton shapes and the target on singleton distractor present trials. A 2 (task type: conjunction search, feature search) × 2 (probe stimulus: color singleton distractor, target-colored shapes pooled) repeated-measures ANOVA was conducted on probe report rate on singleton distractor present trials. The main effects of probe stimulus was significant, *F*(1, 19) = 125.13, *p* < .001, η_p_^2^ = 0.87, BF_10_ > 100. Overall, the singleton probe (13.5%) was reported at a much lower rate than the pooled baseline (42.2%). The main effect of task type was also significant, *F*(1, 19) = 6.19, *p* = .022, η_p_^2^ = 0.25, BF_10_ = 2.43, with slightly higher overall probe report rates in the conjunction search than feature search. Critically, the interaction between task type and probe stimulus was not significant, *F*(1, 19) = 0.75, *p* = .397, η_p_^2^ = 0.04, BF_10_ = 0.39. Probes on the salient distractor were reported at a similar level of reduced rates compared with the pooled baseline in feature search (27.7% below baseline) and conjunction search (29.6% below baseline). This shows that increased search seriality did not affect distractor avoidance in the probe task.

### Discussion

The target in a conjunction search is highly confusable with distractor elements due to their shared component features, and thus has been repeatedly found to be detected inefficiently in previous studies (e.g., Treisman & Gelade, 1980). In the present experiment, the search target was not defined by a single feature as in Gaspelin et al. (2015) or the feature-search conditions in the present study—instead, it was defined based on the spatial arrangement of two features. Despite the extremely low target discriminability and the consequently invoked highly serial search mode, the strength of distractor avoidance was unaffected in both the search and the probe tasks. By manipulating search seriality via top-down task goals, the results support and extend the conclusions of Experiments 1 and 2 that manipulated bottom-up featural differences to reduce search efficiency. The findings contradict the prediction of the attentional window account, but are instead more consistent with the view that distractor avoidance is mediated by top-down attentional control.

## Experiment 4

In Experiments 1–3, search seriality was preliminarily assessed using search efficiency (RT in search trials) and the relative likelihood of reporting probes on the target shape in probe trials. Across all three experiments, search RT was slower and the advantage in reporting target probes was less pronounced in conditions with lower target–distractor discriminability. Although these results were consistent with increased search seriality under our manipulations, they do not unambiguously demonstrate that search operated in accordance with a strict definition of serial inspection. Serial search, as the term implies, involves inspecting items one at a time until the target is located (Treisman & Gelade, 1980). Under this definition, longer RTs and reduced target identification are necessary but not sufficient indicators, because those differences may also arise from other causes—for example, prolonged decision-making or delayed attentional disengagement. Demonstrating truly serial search requires evidence that attention dwells on individual items.

To address this, Experiment 4 manipulated set size (4 vs. 6 items) across all seven search tasks from Experiments 1–3 and examined whether RT increased as a function of the number of items in the search array. The rate of RT increase with set size—the search slope—provides an index of item-based search efficiency (Liesefeld & Müller, 2019; Michaelsen et al., 2024; Palmer, 1995; Townsend, 1971). Steeper slopes are more consistent with item-by-item processing, whereas shallower slopes indicate a more parallel process in which RT is relatively insensitive to set size. If our target–distractor discriminability manipulations increase search seriality, steeper search slopes should be observed under lower discriminability conditions.

### Method

#### Participants

As Experiment 4 serves as a manipulation check for Experiments 1–3, the same sample size was used. A new sample of 24 undergraduate students were recruited. All participants had normal visual acuity and color vision. Informed consent was obtained from each individual.

#### Stimuli, procedure, and design

Experiment 4 included all seven search tasks from Experiments 1–3. Because the primary goal was to examine search slopes, only search trials without color-singleton distractors were included, and the probe task was omitted. The stimuli were otherwise identical to those used in Experiments 1–3. In addition to target–distractor discriminability, the only other independent variable was set size. Each search task included an equal number of trials with four and six items. On set size 4 trials, the displays contained the same four shapes as in Experiments 1–3. On set size 6 trials, two nontarget shapes from the four-item display were randomly selected and duplicated, resulting in six items total. In both set size conditions, stimuli were spaced equidistantly, each positioned 2° from fixation. Previously in Experiments 1–3, stimuli were always presented at the four cardinal locations (top, bottom, left, right). In the present experiment, this could lead those locations to be more strongly associated with the set size 4 condition than the set size 6 condition. To minimize this potential spatial bias, stimulus positions were rotated away from the vertical and/or horizontal axes on half of the trials in both set size conditions.

The order of the three experiments was counterbalanced across participants, with all search tasks within a given experiment administered consecutively, in random order. Target color (red or green) was also counterbalanced across participants and remained constant across all seven search tasks. Each task began with a 16-trial practice block, followed by 72 experimental trials, including 36 trials for each set size condition.

### Results

Participants had to achieve an overall accuracy of 80% or above and a local accuracy of 70% or above in each of the seven search tasks to be included in analysis. Two subjects were removed for their low accuracy. For each of the remaining 22 individuals, trials with incorrect or missing responses (3.9% of total trials) or RTs beyond 2 standard deviations of the mean of each cell condition were not included in RT analysis (4.1% of total trials).

**Fig. 7:**
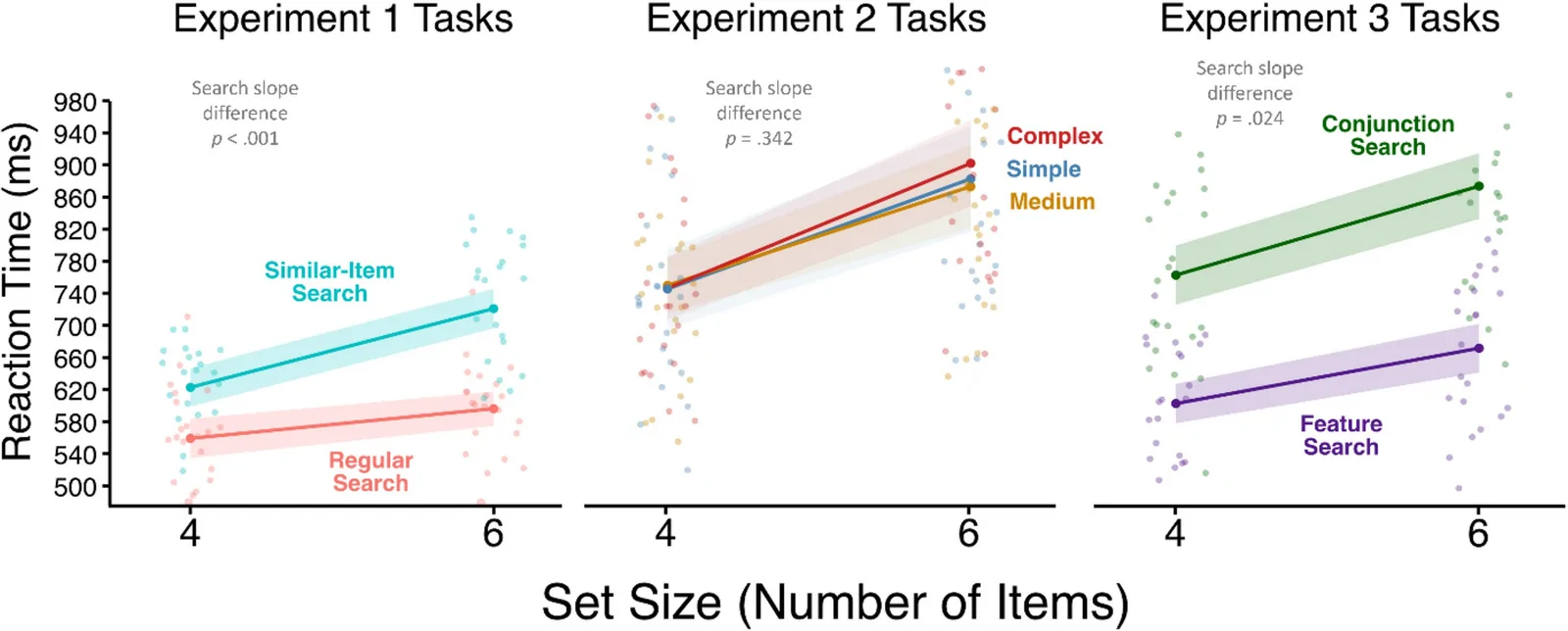
Results of Experiment 4: Reaction time as a function of set size for all seven search tasks used in Experiments 1–3. Search tasks with lower target–distractor discriminability in Experiment 1 (similar-item search) and Experiment 3 (conjunction search) produced significantly steeper search slopes compared with the control conditions, indicating effectively increased search seriality. The complex search of Experiment 2 also produced a numerically steeper, yet not significant, search slope than the easier conditions. Shaded bands represent within-subject standard errors (Cousineau et al., 2021). Scatter dots represent data from individual subjects. (Color figure online)

To assess search seriality under different manipulations, repeated-measures ANOVAs with task type and set size as independent variables were separately performed for each experiment. The RT search slope results are shown in Fig. 7. For Experiment 1 tasks, in RT analysis, the main effect of task type was significant, *F*(1, 21) = 59.67, *p* < .001, η_p_^2^ = 0.74, BF_10_ > 100. RT was slower in the similar-item search than regular search. The main effect of set size was also significant, *F*(1, 21) = 140.38, *p* < .001, η_p_^2^ = 0.87, BF_10_ > 100. RT was slower in set size 6 condition compared with set size 4. Critically, the interaction between task type and set size was significant, *F*(1, 21) = 21.24, *p* < .001, η_p_^2^ = 0.50, BF_10_ > 100. As shown in Fig. 7, the lengthening in RT due to added set size was greater in the similar-item search (98 ms difference; 49 ms/item) than in the regular search (37-ms difference; 18.5 ms/item). That is, the similar-item search showed a steeper search slope than the regular search, confirming the effectiveness of the seriality manipulation of Experiment 1. Analysis of error rate showed consistent results. The main effect of task type was marginally significant, *F*(1, 21) = 4.11, *p* = .056, η_p_^2^ = 0.16, BF_10_ = 1.86, with numerically more errors made in the similar-item search than regular search. The main effect of set size was significant, *F*(1, 21) = 5.74, *p* = .026, η_p_^2^ = 0.22, BF_10_ = 1.92. More errors were made in the set size 6 condition than set size 4. The interaction was marginally significant, *F*(1, 21) = 3.62, *p* = .071, η_p_^2^ = 0.15, BF_10_ = 2.76. The increase in errors due to added set size was numerically greater in the similar-item search (3.3% difference) than regular search (0.3% difference).

For Experiment 2 tasks, in RT analysis, the main effect of task type was not significant, *F*(2, 42) = 0.08, *p* = .921, η_p_^2^ = 0.00, BF_10_ = 0.31. RT barely differed between the three complexity conditions. The main effect of set size was significant, *F*(1, 21) = 202.51, *p* < .001, η_p_^2^ = 0.91, BF_10_ > 100. RT was slower in set size 6 condition compared with set size 4. Unfortunately, the interaction between task type and set size was not significant, *F*(2, 42) = 1.10, *p* = .342, η_p_^2^ = 0.05, BF_10_ = 0.28. The lengthening in RT due to added set size only numerically but not significantly differed between simple (137-ms difference; 68.5 ms/item), medium (123-ms difference; 61.5 ms/item), and complex (155-ms difference; 77.5 ms/item) conditions. Analysis of error rate did not show informative trends regarding search seriality either.^3^ The main effect of task type was not significant, *F*(2, 42) = 0.38, *p* = .689, η_p_^2^ = 0.02, BF_10_ = 8.03. The main effect of set size was significant, *F*(1, 21) = 21.67, *p* < .001, η_p_^2^ = 0.51, BF_10_ = 10.15. More errors were made in set size 6 condition than set size 4. The interaction was not significant, *F*(2, 42) = 0.33, *p* = .722, η_p_^2^ = 0.02, BF_10_ = 42.42. The increase in errors due to added set size only numerically but not significantly differed between simple (2.5% difference), medium (3.8% difference), and complex (2.8% difference) conditions.

For Experiment 3 tasks, in RT analysis, the main effect of task type was significant, *F*(1, 21) = 160.68, *p* < .001, η_p_^2^ = 0.88, BF_10_ > 100. RT was slower in the conjunction search than feature search. The main effect of set size was also significant, *F*(1, 21) = 110.55, *p* < .001, η_p_^2^ = 0.84, BF_10_ > 100. RT was slower in set size 6 condition compared with set size 4. Critically, the interaction between task type and set size was significant, *F*(1, 21) = 5.90, *p* = .024, η_p_^2^ = 0.22, BF_10_ = 16.18. As shown in Fig. 7, the lengthening in RT due to added set size was greater in the conjunction search (111-ms difference; 55.5 ms/item) than in the feature search (69-ms difference; 34.5 ms/item). That is, the conjunction search showed a steeper search slope than the feature search, confirming the effectiveness of the seriality manipulation of Experiment 3. Analysis of error rate showed consistent results. The main effect of task type was significant, *F*(1, 21) = 31.06, *p* < .001, η_p_^2^ = 0.60, BF_10_ > 100, with more errors made in the conjunction search than feature search. The main effect of set size was significant, *F*(1, 21) = 6.08, *p* = .022, η_p_^2^ = 0.22, BF_10_ = 5.25. More errors were made in set size 6 condition than set size 4. The interaction was significant, *F*(1, 21) = 4.78, *p* = .040, η_p_^2^ = 0.19, BF_10_ = 9.61. The increase in errors due to added set size was numerically greater in the conjunction search (2.7% difference) than feature search (0.1% difference).

### Discussion

Experiment 4 aimed to provide additional evidence for an item-by-item serial search process under our target–distractor discriminability manipulations from Experiments 1–3. The presumably more serial conditions from Experiments 1 and 3 both produced significantly steeper search slopes than the control conditions. These results confirmed the effectiveness of varying stimulus physical resemblance and conjunctive search demands to invoke serial search modes.

In contrast, the stimulus complexity manipulation from Experiment 2 did not produce graded differences in search slopes, although the comparison between simple and complex conditions showed a numerical increase in the expected direction. Notably, there was also little evidence that participants were sensitive to the complexity manipulation in the first place. Unlike in Experiment 2, the main effect of task type was not significant in the present experiment, indicating that responses were not reliably slowed by increased complexity. Thus, the absence of search slope differences may reflect a general insensitivity to the complexity manipulation for these participants. Other factors may also have contributed to the null effect. For instance, the irregular stimulus shapes used in Experiment 2 created more visual heterogeneity than the geometrically regular shapes of Experiments 1 and 3. In Experiment 4, the larger set size increased display crowding, and the removal of consistent cardinal locations further reduced spatial regularity. These manipulations may therefore have disproportionately affected processing of the irregular-shape displays from Experiment 2 relative to the more regular displays in Experiments 1 and 3. The difficulty in processing a crowded, highly heterogeneous display may have obscured any effects of stimulus complexity on search slopes. Thus, the results of the present experiment appear inconclusive with respect to whether stimulus complexity, as manipulated in Experiment 2, affected search seriality.

## General discussion

The present study used three different approaches to decrease the discriminability of the target in a search array, presumably increasing search seriality—and examined the effect of the increased seriality on avoidance of salient distractors. Experiment 1 manipulated physical resemblance by presenting a target circle among physically similar high-sided polygons to reduce its discriminability. Experiment 2 manipulated representational difficulty by using irregular shapes with different numbers of vertices. Shapes with more vertices, and thus more complexity, were less identifiable amongst each other. Experiment 3 manipulated top-down factors by imposing conjunction-search requirements, where identifying the target required attention to more than one feature. The target–distractor discriminability manipulations were preliminarily proven to be effective based on two indicators within the three experiments: Participants demonstrated reduced search efficiency and reduced probe report rates for the target in the conditions with low target discriminability. Although sensitive to the manipulations, participants’ ability to avoid distraction by a salient distractor was unaffected: In all three experiments, attentional allocation avoided a color-singleton distractor, with the magnitude of the distractor avoidance effect unaffected by the discriminability manipulation. In addition, a control experiment provided converging evidence that the methods used in Experiments 1 and 3 elicited serial search processes involving item-by-item inspection of the array, while the method of Experiment 2 produced a numerical but not significant trend of increased seriality. Taken together, the results suggest that distractor avoidance is not due to passive exclusion, a claim of the attentional window account, and instead it is more likely due to active goal-based control of attention that favors relevant items and avoids irrelevant ones.

### Implications for the mechanisms of distractor avoidance

The present findings shed light on the debate over the manner in which disruptions by bottom-up salience can be prevented. On the one hand, theories of top-down control state that attention can be guided to avoid distractors that do not possess the target features. Extending the basic theories of feature guidance, the signal suppression hypothesis further proposed that distractor features that signal salience can be actively downweighted (Gaspelin et al., 2015; Sawaki & Luck, 2010). On the other hand, the attentional window account argues that distractor avoidance is a consequence of attention being narrowly focused elsewhere in a highly serial search (Theeuwes, 2004, 2023). The theory argues that a small “attentional window” is adopted during a serial search that requires fine discrimination, rendering salient signals that fall outside of the focused attentional window passively excluded from selection. If the attentional window account is correct, the smaller the size of the attentional window, the less likely a distractor will be selected for processing (see additional discussion in Gaspelin, Egeth, et al. 2023). To test this prediction, the present study created different levels of seriality in visual search tasks. Experiments 1–3 separately increased the physical resemblance, increased the representational distinction between the target and the nontargets, or required binding of multiple features to identify the target. These manipulations aimed to create highly serial search processes, which according to the attentional window account should increase the chance for the salient distractor to fall outside of the narrow attentional focus and consequently facilitate stronger distractor avoidance. However, the absence of differences in distractor avoidance between the highly serial search conditions and the control conditions in all three experiments violates the prediction of the attentional window account. Instead, the present study provides converging evidence that distractor avoidance is not contingent on the seriality of the search process. The results are incompatible with the passive exclusion explanation of distractor avoidance, but more consistent with an active control mechanism.

### Limitations in supporting evidence for the attentional window account

Initial evidence for the attentional window account was put forth by Belopolsky and colleagues (2007). They had participants search for a target letter among an array of letters, with one of the letters (sometimes the target) presented in a unique color. The search was preceded by a task in which the breadth of attention was manipulated: In a *diffuse* condition, participants identified the shape of the global array, and in a *focused* condition they identified the shape at central fixation. The shapes served as go/no-go signals, indicating whether participants should then proceed with the search. The diffuse/focused manipulation was presumed to have invoked attentional windows either enclosing or excluding the uniquely colored distractor shape in the display. Belopolsky and colleagues (2007) found that the color singleton only captured attention in the diffuse condition but not in the focused condition, and they therefore concluded that salient distractors inside the attentional window cannot be ignored.

There are several reasons to question the extent to which Belopolsky et al.’s (2007) conclusions apply to visual search more generally. First, in the diffuse condition, when subjects were required to determine the shape of the elements in the display—that shape included the color singleton. Thus, participants were actually *required* to process the singleton on such trials. However, in the focused condition, participants attended to a small shape at fixation and were not required to process the singleton. That difference alone might account for the differential effects of the color singleton in the two conditions. Additionally, the color singleton was the target on some trials, which might have encouraged inspection of the singleton. Recently, a study using similar methods as Belopolsky et al. (2007) failed to replicate the findings (Ruthruff et al., 2025).

### Search mode versus attentional window

As previously discussed, other evidence in support of the attentional window account has been offered from studies that employed the additional singleton task (e.g., Theeuwes, 2004). In that task participants look for a unique shape among homogeneous shapes, sometimes in the presence of a uniquely colored distractor. The assumption is that such tasks cause participants to adopt a wide attentional window in order to efficiently find the unique shape. And the fact that the distractor leads to performance decrements in such cases has been used to argue that the salient distractor automatically captured attention because it fell within the (presumably wide) attentional window. Nevertheless, an alternative interpretation is that the requirement to locate a unique shape is likely to cause participants to adopt a search mode in which they are specifically looking for unique things (singleton detection mode; Bacon & Egeth, 1994). As a result, a uniquely colored distractor may also be attended. However, in that case, attentional capture by the singleton would not be attributable to its bottom-up salience—instead, it seems likely to have been driven by the top-down search goal. As a result, evidence of attentional capture from additional singleton tasks must be interpreted with caution.

Leber and Egeth (2006) were one of the first to attempt to distinguish the impact of different search modes from the effects of changing the size of the attentional window. In their study, in an initial training phase, one group of participants performed a feature-search task in which they searched for a specific shape amongst heterogeneous shapes; another group performed an additional singleton task, with the unique target shape changing unpredictably across trials. In a subsequent phase of the experiment, both groups performed a modified additional singleton task in which the unique target was the same shape on every trial. Thus, this task could have been performed either by searching for a specific shape (feature-search mode; which one group had developed through training) or by searching for the unique item (singleton detection mode; which the other group had developed). Distinct effects were found in the two groups: Participants trained on the additional singleton task were distracted by the color singleton, but participants trained on the specific-target search were not. Importantly, the search slopes (RT as a function of display size) were flat in both groups, suggesting a near-parallel search and a large attentional window. Thus, the participants were able to ignore the salient distractor inside the large attentional window. Attentional capture in the group of participants trained on the additional singleton task can thus be attributed to the singleton-seeking top-down goal.

### Past evidence challenging the attentional window account

Research suggests that distractor ignoring in many situations is contingent on knowing the feature of the salient distractor (for an exception see Ma & Abrams, 2023a, b), rather than contingent on a narrow attentional breadth. Several studies have shown that salient distractors of unknown features cannot be ignored even when attention is narrowly focused (Gaspelin & Luck, 2018b; Oxner et al., 2023; Vatterott et al., 2018). For example, Vatterott et al. (2018) had the distractor appear in a fixed color within a block, but changed it to a new color in every new block. Split-half analysis of the blocks showed attentional capture by the novel-color singleton in the first block halves, but the capture effect was eliminated in the second block halves. With distractor ignoring shown to be possible in the second block halves, the task should invoke a small attentional window according to the attentional window account. However, novel distractor colors nevertheless could not be overlooked. These results suggest that distractor ignoring relies on active top-down guidance based on the distractor features, irrespective of the size of the attentional window.

As an alternative to manipulating the seriality of search to test the attentional window account, in an EEG study, Ma et al. (2026) explicitly manipulated the size of the attentional window. Participants were asked to report the shape of either a central, inner contour or a peripheral, outer contour of the display. In between the two contours, an array of homogeneously colored disks and a uniquely colored distractor were presented. Focusing attention on the inner contour excluded the salient distractor from the attentional window, while expanding attention to identify the outer contour included the distractor within the attentional window. An additional control condition was conducted, where participants were required to attend to the singleton distractor and report a feature on it. The results showed that neither the focused nor the expanded conditions produced an N2pc component contralateral to the singleton distractor—a response that would be expected if the distractor had captured attention (Eimer, 1996; Hopf et al., 2000; Luck & Hillyard, 1994). In contrast, the singleton-search control condition produced a significant N2pc, verifying the sensitivity of the paradigm to detect attentional allocation to the distractor. This elimination of capture by salient distractors within the breadth of attention runs counter to the core idea of the attentional window account, and is thus consistent with a feature-based control mechanism independent of the focus of attention.

Additionally, there is evidence suggesting that, in many search tasks, attention is broadly spread at the moment of search array onset, rather than narrowly focused as assumed by the attentional window account in cases in which distractor avoidance occurs. In other words, the searches that permit distractor avoidance are not as serial as presumed. For example, in the search for a specific target shape (as shown in Fig. 1a) that might be thought to require a small attentional window, the target is actually highly likely to be promptly attended as soon as the display is presented: Based on the probe-task results from several experiments (Gaspelin et al., 2015; Ma & Abrams, 2023c; Stilwell et al., 2023; Stilwell & Gaspelin, 2021), the search target had a significantly higher probe report rate (e.g., an average of 52.6% across Experiments 2–4 in Gaspelin et al., 2015) than the nonsingleton shapes (e.g., an average of 24.4% across Experiments 2–4 in Gaspelin et al., 2015). Eye-tracking studies also showed that the first saccade accurately landed on the target shape at a likelihood significantly higher than chance (Drennan & Gaspelin, 2026; Gaspelin et al., 2017; Zhang & Gaspelin, 2026). Nevertheless, participants in those tasks did indeed avoid capture by the salient distractor. If the searches in those studies simply involved a highly serial search with a small attentional window, then the initial shift of attention should be relatively random, rendering the target and the target-colored nonsingleton shapes equally likely to be selected. The observed bias toward the target after a very brief exposure suggests instead that a considerable amount of global processing of the array occurs prior to a shift of attention to the target. Such global processing would presumably not exclude the saliency signal of a distractor. Therefore, it is unlikely that search seriality really accounts for distractor ignoring. In the present study, we created highly serial search conditions that presumably satisfy the requirements of the attentional window account, yet we found no facilitation of distractor avoidance. The avoidance of the distractor must alternatively be achieved by active control mechanisms.

### Multiple control mechanisms contributing to distractor avoidance

It is worth clarifying that while we have interpreted the present results as supportive of the role of top-down control in distractor avoidance, the observed effects may have been achieved through mechanisms other than distractor downweighting. Guided by task goals, attention prioritizes relevant items while avoiding irrelevant items, and this process may involve multiple feature-based mechanisms (e.g., distractor feature inhibition, biased competition, or target feature enhancement; Chang & Egeth, 2019; Desimone & Duncan, 1995; Gaspelin et al., 2025; Khodayari et al., 2026; Wolfe, 2021). For example, previous studies have shown that the prevention of attentional capture by salient distractors may be due in part to prioritization of target-matching features (Chang & Egeth, 2019, 2021; Oxner et al., 2023). In those studies, when participants were searching for a prespecified target (e.g., in green) while avoiding a singleton distractor (e.g., in red), target-colored nonsingleton items (e.g., green shapes) were shown to receive enhanced processing relative to novel-colored nonsingleton items (e.g., blue shapes). Such target feature enhancement may also have contributed to the distractor avoidance effect in the present study. Thus, while our findings are broadly consistent with top-down control accounts of distractor avoidance, they do not uniquely implicate distractor inhibition over other feature-based mechanisms, such as target enhancement or biased competition. Importantly, all these mechanisms rely on active control based on top-down goals and/or selection history, which is fundamentally different from the nonvolitional filtering proposed by the attentional window account.

### Limitations of the present study

It is worth noting that the search tasks serving as control conditions in the present study (e.g., regular search in Experiment 1 and feature search in Experiment 3) have been characterized as highly serial under the attentional window account as they permit distractor ignoring (Theeuwes, 2023). If correct, distractor avoidance in those conditions may already be near ceiling and therefore unlikely to increase further as the search becomes more serial. However, as discussed earlier, multiple lines of evidence suggest that these searches may not be as strictly serial as previously assumed. Behavioral and eye-tracking studies indicate substantial preattentive global processing prior to the first attentional shift (e.g., Gaspelin et al., 2015, 2017). Moreover, in Experiment 4, which manipulated set size, the baseline search tasks (from Experiments 1 and 3) exhibited much shallower search slopes compared with the more serial conditions. Together, these findings suggest that the control tasks do not fully instantiate strictly serial search, contrary to the assumptions of the attentional window account (Theeuwes, 2023). Recent formulations of the theory also allow for intermediate search modes, such as “clump-wise” processing (de Waard & Theeuwes, 2026), which blurs the strict distinction between serial and parallel search by permitting the attentional window to vary with the size of grouped items. The previously used search tasks that permit distractor ignoring as well as the control conditions in the present study likely only invoke clump-wise search modes with medium-sized attentional windows. This perspective partly motivated the present study, which aimed to induce more typical item-by-item serial search processes in order to better evaluate the possibility of spatially excluding distractors from a truly narrow attentional window.

An alternative approach to testing the attentional window account would be to manipulate search toward more parallel modes rather than more serial ones, particularly given that distractor avoidance was already observed in the present control conditions. This would provide a complementary test of competing accounts of passive filtering versus active control, and thus a meaningful future direction. Alternatively, instead of manipulating search seriality, one could directly vary the size of the attentional window by requiring inspection of a broader region of the display. For example, a recent study adopted this approach by encouraging a broad attentional window that encompassed a salient distractor and found that distractor suppression could still be observed (Ma et al., 2026). This provides preliminary evidence that a more parallel search mode may nevertheless not disable distractor avoidance, which is inconsistent with the predictions of the attentional window account.

Finally, some recent research has pointed out that letter probe reporting methods like that used in the present experiments are subject to the influence of observer’s bias (Kerzel & Renaud, 2023). With the task instruction of ignoring the salient distractor, it is possible that the letters that the observer chose to report are influenced by demand characteristics. This concern is particularly amplified by the fact that only a small subset of the presented letters was selectively reported on each trial, and the letters were recalled from memory after a delay. The reason that such decision-level bias may concern the present study is that if the observed probe suppression effect is completely due to demand characteristics, the magnitude of the effect would be expected to remain constant regardless of search seriality, which is exactly what we observed. As an attempt to evaluate distractor avoidance in a bias-free way, Ma and Abrams (2025) assessed the magnitude of suppression by examining the impact of target–distractor congruency on the identification of targets, with a greater congruency effect suggesting more attention allocated to the distractor. Using this method, that precludes any decision-level biases, significant distractor suppression was still present, with the pattern of results very similar to that obtained using the probe method. This suggests that the probe suppression effect at least in part reflects true perceptual-level distractor avoidance, and the probe method can still provide useful information about attentional allocation to individual stimuli.

## Conclusion

Two competing explanations have been proposed to explain the manner in which attentional capture by salient distractors can be avoided in visual search: The top-down control account proposes that attention is actively guided to avoid irrelevant distractor features; the attentional window account proposes that distractors are passively excluded from a narrow attentional focus under a highly serial search. The present study created that highly serial search condition that was not available in previous studies, and tested the core prediction of the attentional window account that the strength of distractor avoidance would depend on the degree of search seriality. Three experiments successfully manipulated search seriality, yet the extent to which salient distractors were avoided was unaffected. The findings are inconsistent with the core idea of the attentional window account, and thus imply that top-down control is a more likely mechanism for distractor avoidance.

## Data Availability

All data has been made publicly available (https://osf.io/njvu2).

## Footnotes

1 The statistical conclusions drawn from the Bayesian and NHST analyses were inconsistent for this effect. However, both results appear to be near the threshold of significance, and this main effect is not critical to the primary purpose of the experiment.

2 It is not necessary for the purpose of the present experiment to have the color strips vertically aligned in the singleton distractor. We intended to make the singleton distractor salient by its uniqueness in color, rather than its uniqueness in orientation. Indeed, uniqueness in color has been shown to be perceived as more salient and have a higher likelihood of causing capture than uniqueness in the configuration of a shape (Theeuwes,1992). This design, however, was used in preparation for future experiments on suppression of distractors of unpredictable colors (i.e., second-order suppression). In those situations, the salient distractor randomly swaps color with the rest of the array, and thus needs to be distinguishable from the remaining elements based on a feature other than color.

3 In the error rate analysis, the Bayesian and NHST results were inconsistent for a few effects. However, these effects were not critical to the interpretation of the findings and were not numerically large enough to warrant additional consideration.

## Code availability

All analysis code has been made publicly available (https://osf.io/njvu2).
